# Kinetics of the Toluene Reaction with OH Radical

**DOI:** 10.34133/2019/5373785

**Published:** 2019-05-29

**Authors:** Rui Ming Zhang, Donald G. Truhlar, Xuefei Xu

**Affiliations:** ^1^Center for Combustion Energy, Department of Energy and Power Engineering, and Key Laboratory for Thermal Science and Power Engineering of Ministry of Education, Tsinghua University, Beijing 100084, China; ^2^Department of Chemistry, Chemical Theory Center, and Minnesota Supercomputing Institute, University of Minnesota, Minneapolis, MN 55455-0431, USA

## Abstract

We calculated the kinetics of chemical activation reactions of toluene with hydroxyl radical in the temperature range from 213 K to 2500 K and the pressure range from 10 Torr to the high-pressure limit by using multistructural variational transition state theory with the small-curvature tunneling approximation (MS-CVT/SCT) and using the system-specific quantum Rice-Ramsperger-Kassel method. The reactions of OH with toluene are important elementary steps in both combustion and atmospheric chemistry, and thus it is valuable to understand the rate constants both in the high-pressure, high-temperature regime and in the low-pressure, low-temperature regime. Under the experimental pressure conditions, the theoretically calculated total reaction rate constants agree well with the limited experimental data, including the negative temperature dependence at low temperature. We find that the effect of multistructural anharmonicity on the partition functions usually increases with temperature, and it can change the calculated reaction rates by factors as small as 0.2 and as large as 4.2. We also find a large effect of anharmonicity on the zero-point energies of the transition states for the abstraction reactions. We report that abstraction of H from methyl should not be neglected in atmospheric chemistry, even though the low-temperature results are dominated by addition. We calculated the product distribution, which is usually not accessible to experiments, as a function of temperature and pressure.

## 1. Introduction

**Figure 1:**
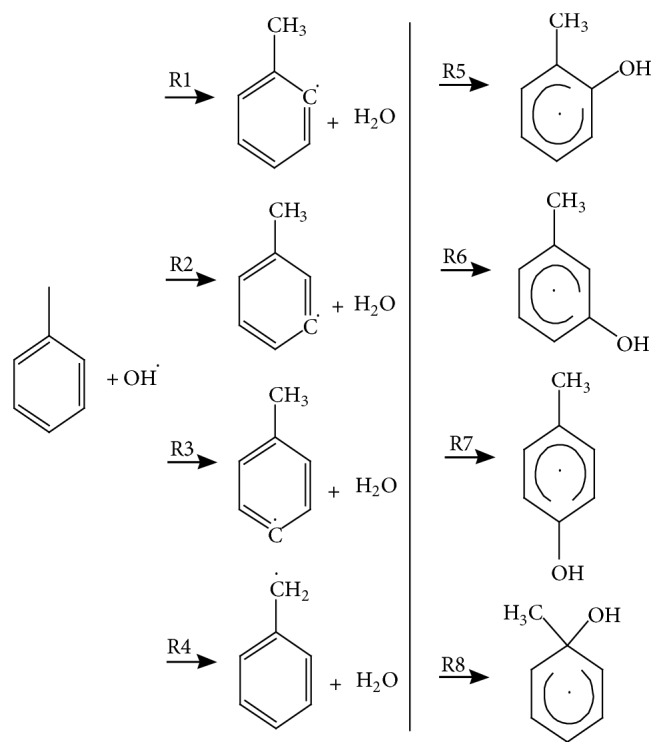
The reactions whose rate constants are calculated in this work.

Aromatic hydrocarbons are important in both combustion chemistry and atmospheric chemistry; they are also major petrochemicals, and they are used in synthesis, as solvents, and as fuel additives. They are known to play a major role in emission and soot formation (particulate polycyclic aromatic hydrocarbons) during fossil fuel combustion and in the formation of photochemical smog in the urban air [1, 2]. Therefore, the oxidation mechanisms and reaction kinetics of aromatic hydrocarbons are of great interest over wide pressure and temperature ranges. Toluene is one of the simplest aromatic hydrocarbons, and its reaction with the OH radical, a main oxidizing reactive species in both combustion and the atmosphere, has been extensively investigated both experimentally and theoretically [3–13]. The main reaction channels of toluene with OH radical are hydrogen abstraction reactions (R1-R4) and addition reactions (R5-R8), as shown in Figure 1. (Note that reactions R5–R8 in this figure involve the formation of bonded adducts, but the figure does not show the pre-reactive van der Waals complex of OH with toluene. We assume a fast pre-equilibrium between the reactants and this complex, and this has an effect on the tunneling calculations for the hydrogen abstraction reactions [11].)

The first experimental study of the kinetics of the OH + toluene reaction was carried out by Davis et al. [3] at 298 K and 3–100 Torr total pressure; they found the rate constant to be pressure-dependent, which they interpreted as showing that a significant amount of reaction occurs by addition to the ring. Additional experiments were presented by Doyle et al. [4] (304 K, 1 atm), Hansen et al. [5] (298 K, 100–618 Torr), and Perry et al. [6] (298–473 K, 100–200 Torr), where the values in parentheses are ranges of temperature and total pressure. Neither Hansen nor Perry found significant pressure dependence. For temperatures between ~325 K and 380 K, Perry et al. found nonexponential decay of the total rate constant, indicating reversible reaction; hence their analysis is not valid at those temperatures.

The most complete set of available experiments for OH + toluene kinetics are those of Tully et al. [7] (213–1150 K, 20–200 Torr). They analyzed their experiments under the assumption that the loss of OH followed pseudo-first-order kinetics without back reaction, but at temperatures in the interval 320–352 K, the loss of OH showed nonexponential decay, indicating that the assumption was not valid and that an accurate rate constant cannot be obtained from their experiments at those temperatures. Furthermore, at temperatures from 352 to 442 K, their extracted pseudo-first-order rate constants depended linearly on the concentration of toluene only over a small concentration interval at low concentrations of toluene and the measured rate constants depended on the initial OH concentration, so the extracted rate constants have large uncertainties in this range. Tully et al. concluded that the observations of nonexponential decays and the nonlinear concentration plots “cannot satisfactorily be explained at this time.” However, they did interpret some of their observations in terms of reversible reaction, i.e., decomposition of the newly formed toluene…OH adducts back to reactants. Based on these observations, only their experimental results in the temperature intervals not affected by these problems are meaningful for comparison with our theoretical estimates of the elementary-reaction rate constant for the forward reaction; these temperature intervals are 213–298 K and 504–1150 K.

Seta et al. [8] measured the reaction rate at 919–1481 K (at 0.8–1.8 atm), where it is essentially all due to abstraction reactions. Vasudevan et al. [9] reported the reaction constant between 911 K and 1389 K at the pressure of ~2.25 atm. Their rate constants range from 13% lower than those of Seta et al. at 919 K to 39% lower at 1389 K.

Uc et al. [10] used transition state theory (TST) with the Eckart approximation to zero-curvature tunneling to calculate the rate constants of the hydrogen abstraction reactions at 275–1000 K and the overall rate constants (taking into account all possible abstraction and addition reactions) at 200–600 K; they used a potential energy surface calculated by CBS/QB3//BHandHLYP/6-311++G(d,p). They included torsional anharmonicity by a one-dimensional approximation. Seta et al. [8] also estimated rate constants using TST with the Eckart approximation to zero-curvature tunneling, but in their case with B3LYP/6-31G(d) potential energy surfaces on which they adjusted the barrier heights to obtain agreement with experiment at high temperature, and they estimated the effects of anharmonicity originating from the –OH torsion and the C-H-O rocking. Li et al. [12] used TST with the Wigner tunneling approximation with G4//B3LYP/6-31G(2df,p) potential energy surfaces to estimate the rate constants for the abstraction paths; they do not mention including anharmonicity in the rate calculation. Recently, Pelucchi et al. [13] reported more reliable calculations of the rate constants for the abstraction reactions by employing canonical variational transition state theory (CVT) with the Eckart approximation to zero-curvature tunneling and with the potential energy surface calculated by M06-2X/6-311+G(d,p) and CCSD(T)/aug-cc-pVTZ with a correction for basis set effects. In their calculations, they considered the effect of torsional anharmonicity on the rate constants by using a two-dimensional hindered rotor method for hydrogen abstraction from the methyl group.

The above summary shows that the H-abstraction reactions have been studied much more than the pressure-dependent addition reactions. The addition of OH to toluene is similar in many respects to the addition of OH to benzene, which was also studied in some of the papers cited above, as well as by several other workers. Lin et al. included the reversible reaction in an analysis of the OH + benzene problem and were able to obtain the equilibrium constant for the formation of the benzene...OH adduct as well as the forward and backward rate constants for its formation [14]. In later work, Uc et al. reanalyzed the experiments of Tully et al. on benzene and toluene with the help of theoretical equilibrium constants for adduct formation [11]. The calculations indicated that back reaction of the adduct becomes increasingly important at temperatures of 325 K and greater, and they were able to explain the signals observed by Tully et al. in terms of the contribution of the reverse reaction. This complication in the kinetics of the addition reaction does not affect the measured results at high temperature where the abstraction channel dominates the total reaction.

The work of Uc et al. stressed the role of the reverse dissociation reaction in leading to nonexponential decay of the OH concentration. This motivates explicit consideration of the pressure effect because the unimolecular dissociation reaction of the adduct is pressure-dependent.* As far as we know, no theoretical study has been reported on the pressure effects on the toluene plus OH reaction.* Experimentally, the pressure effect has mainly been studied at 298 K. As mentioned above, Davis et al. [3] found the rate constant to be pressure-dependent at 298 K and 3-100 Torr total pressure; but neither Hansen [5] nor Perry [6] found significant pressure dependence at 298 K and above 100 Torr. Tully et al. [7] reconfirmed their work by finding that the third-body dependence was only 9% at 298 K and 100 Torr in He, Ar, or SF_6_; they concluded that the experiments at 298 K and over 100 Torr are close to the high-pressure limit. Lowering the pressure to 25 Torr in Ar lowered the rate by 10%, but the experimental uncertainty in the rate constants is 11% for that case. Lowering the pressure to 20 Torr in He lowered the rate by 21%, which is significant compared to the 10% experimental uncertainty in the rate constants for that case. With increasing temperature, the pressure fall-off of the addition reaction rate constant should be larger, although systematic study of the effect of pressure on rate constants for* T* > 298 K is missing. Therefore, it is interesting to explore the pressure dependence quantitatively.

In the present work, we carried out a theoretical study of kinetics of the toluene reaction with OH that includes the pressure effects.

## 2. Electronic Structure Calculations

An accurate potential energy surface is the foundation of reliable dynamics calculations. Although generally reliable wave function methods are available for small, weakly correlated systems, it is impractical to use them in direct dynamics calculations for medium- or large-size systems due to the high computational cost. A more practical scheme is to choose a density functional for a Kohn-Sham (KS) model chemistry for the specific system by benchmark calculations of the stationary points on the potential energy surface of that system.

We will use the following notation: Born-Oppenheimer potential energies are electronic energies (including nuclear repulsion) calculated for fixed nuclear coordinates. The classical energy of reaction* ΔE is* defined as the Born-Oppenheimer potential energy of the lowest-energy equilibrium structure of products minus the Born-Oppenheimer potential energy of the lowest-energy equilibrium structure of reactants; the classical barrier height* V* ^‡^ is defined as the Born-Oppenheimer potential energy of the lowest-energy saddle point minus the Born-Oppenheimer potential energy of the lowest-energy equilibrium structure of reactants. Adding the change in zero-point energy (products minus reactants) to the energy of reaction yields the enthalpy of reaction at 0 K. Adding to the classical barrier height the change in zero-point energy on proceeding to the transition state from the reactants yields the enthalpy of activation at 0 K. The temperature-dependent Arrhenius activation energy is obtained from the local slope of an Arrhenius plot of the logarithm of a rate constant versus the reciprocal of the temperature.

*Gaussian 09* [15] and a locally modified version of* Gaussian* [16] were used for the density functional calculations.

### 2.1. Best Estimates of Reaction Energetics

To find and validate a successful model chemistry for present system, we first calculated the geometrical structures of the stationary points (reactants, transition structures, and products) on the potential energy surface by using the M08-HX/MG3S method, which has been recommended for locating transition state structures in a previous benchmark study [17]. Subsequent frequency calculations using the same method confirmed that the structures we obtained are the desired minima and transition structures. Then we use these geometries to calculate the* T*_1_ diagnostics by the CCSD [18] /jun-cc-pVTZ [19–22] method. The standard assumptions are that closed-shell species with *T*_1_>  ~0.02 and open-shell species with *T*_1_>  ~0.045 are strongly correlated and need to be treated with a multireference method, whereas systems with lower* T*_1_ diagnostics are weakly correlated and can be treated reliably by a single-reference method. As shown in Table 1, the obtained* T*_1_ values are all smaller than these borders between small and moderate multireference character. This implies that we can use the single-reference CCSD(T)-F12a [23, 24] /jun-cc-pVTZ method, a highly accurate method for weakly correlated systems, to obtain the best estimates of the classical energies of reaction and barrier heights and then use them to validate the KS model chemistry.

*Molpro 2012.1* [25] was used for the coupled cluster calculations.

### 2.2. Selection of KS Model Chemistry

In our experience, the M06-2X [26], M08-SO [27], and MN15 [28] density functionals combined with the MG3S [29] basis set perform well in calculations of potential energy surfaces for many reaction systems. Therefore, we tested the performance of these three KS model chemistries against the best estimates, and we selected the most accurate of them to be used for direct dynamics calculations for hydrogen abstraction reactions and addition reactions.  Table 2 lists the calculated classical energies of reaction (Δ*E*) and the forward (*V*_*f*_^‡^) and reverse (*V*_*r*_^‡^) barrier heights using these KS model chemistries and compares them to the best estimates obtained by CCSD(T)-F12a/jun-cc-pVTZ//M08-HX/MG3S; the table also gives the mean unsigned derivations (MUDs) of these KS results from the best estimates.

As shown in Table 2, the M06-2X/MG3S method performs best for hydrogen abstraction reactions with a mean unsigned deviation (MUD) of 0.62 kcal/mol over all classical barrier heights and reaction energies of the abstraction reactions, and the M08-SO/MG3S model chemistry is the best choice for addition reactions with a MUD of 0.58 kcal/mol over all classical barrier heights and reaction energies of the addition reactions. Therefore, these two model chemistries were chosen for the direct dynamics calculations of the corresponding temperature-dependent reaction rate constants.

**Figure 2:**
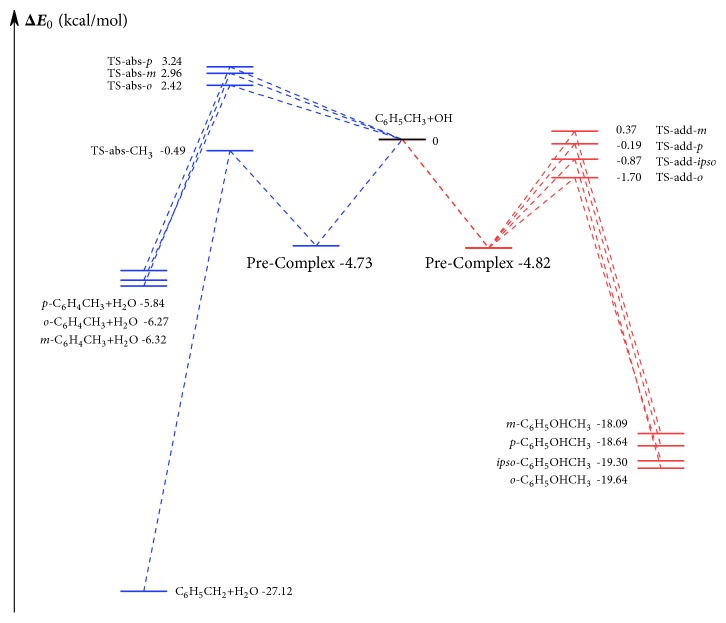
Enthalpy of activation profiles at 0 K for the toluene reaction with OH, as calculated by the M06-2X/MG3S method for hydrogen abstraction channels and by the M08-SO/MG3S method for OH-addition channels. Enthalpy of activation profiles at 0 K includes the zero-point energy, which is calculated by the quasiharmonic approximation with frequencies scaled with the SRP scaling factors.

The enthalpy of activation profiles at 0 K are shown in Figure 2.

### 2.3. Decomposition of Adducts

Previous work [8, 30] on radical addition to toluene has sometimes considered the reactive decomposition of adducts. In the present case we estimated the enthalpies of activation for decomposition of the adducts to make phenol + methyl radical are 4.7–8.5 kcal/mol (at 0 K) higher than the enthalpies of activation for nonreactive reversion to reactants. This agrees with the general trend found by Seta et al. [8]. Therefore these reactions are not major pathways, and we did not consider them further.

## 3. Dynamics Calculations

For dynamic calculations we mainly used the same methods as those used for toluene + H reactions by Bao, Zheng, and one of the current authors [30]. In particular, for the temperature-dependent high-pressure-limit (HPL) rate constants we used multistructural [31] canonical variational transition state theory (CVT) [32] with small-curvature tunneling [24, 33]. As usual, we denote this method as MS-CVT/SCT. In CVT, the transition state is variationally located at the position of maximum free energy of activation along the minimum energy path [34]. In the later discussion we shall refer to the difference between CVT and conventional transition state theory (TST, in which the transition state passes through the saddle point) as a variational-transition-state-location effect or sometimes simply as a variational effect.

In these calculations, we used the multistructural torsional anharmonicity method (MS-T) [35] to treat the multistructural anharmonic effects, by which we mean the torsional potential anharmonicity, the multiple conformers, and the torsion-rotation coupling. We used specific-reaction-parameter (SRP) scaling factors [36, 37] to scale all calculated vibrational frequencies of all species (reactants, pre-reactive van der Waals complex, transition structures, and products); these factors are explained in more detail below. When we use scaled frequencies with the harmonic oscillator formulas for partition coefficients, the result is called quasiharmonic; we also used scaled frequencies in the MS-T calculations. The ratio of the MS-T partition function to the quasiharmonic one for species *α* is called *F*_*α*_^MS-T^.

The pressure-dependent rate constants of the addition reactions were calculated by the system-specific quantum Rice-Ramsperger-Kassel [30] (SS-QRRK) method.

The MS-CVT/SCT method, reaction-specific scale factors, and the SS-QRRK method used in the present study have been described in previous papers [30, 38–40]. Here, we only briefly summarize them.

### 3.1. MS-CVT/SCT Theory

The temperature-dependent high-pressure-limit rate constants calculated by the MS-CVT/SCT theory are expressed by

(1a) kMS-CVT/SCT=kCVTTκSCTTFactMS-TT

(1b) kCVT=ΓTkTSTT

where *k* ^CVT^ is the CVT reaction rate constant obtained in the single-structure quasiharmonic oscillator approximation, Γ is the recrossing transmission coefficient (also called the variational-transition-state-location effect or the variational effect) that accounts for recrossing of the conventional transition state by calculating the flux through a dividing surface corresponding to the maximum free energy of activation, rather than a dividing surface corresponding to the conventional transition state, *k* ^TST^ is the conventional transition state theory rate constant, *κ* ^SCT^ is the calculated tunneling transmission coefficient in the small-curvature tunneling approximation (although it is called the tunneling transmission coefficient, it also includes nonclassical reflection at energies over the barrier top), and *F*_act_^MS-T^ is defined [41] as the multistructural anharmonicity factor of the reaction. The *F*_act_^MS-T^ factor is calculated as the ratio of multistructural anharmonicity factor *F*_*α*_^MS-T^ of the transition state (*α* = TS) to the multistructural anharmonicity factor of the reactant (*α* = R).

In the calculation of *k* ^CVT^, only the lowest-energy structures of the reactants and transition states are used, even when more than one conformer is located for the species. The higher-energy conformers are accounted for by *F*_act_^MS-T^(*T*).

In the high-pressure limit the pre-reactive complex (an OH…toluene van der Waals complex) is fully equilibrated and thermalized, so we took the ground-state energy of this complex as the lowest-energy at which the tunneling occurs. This is called the pre-equilibrium model (PEM). This model was explained in detail in a recent study of the methanol reaction with OH radical [42].

The standard vibrational frequency scaling factor [36] *λ* ^ZPE^ is parametrized to reproduce the accurate zero-point energies (ZPEs) of a set of stable molecules, and this scaling factor is used for reactants, pre-reactive van der Waals complexes, and products, but previous work on transition states of some hydrogen abstraction reactions by OH radical [36, 42] has indicated that this scaling factor is not suitable for such transition states. Therefore, we used a specific-reaction-parameter scaling factor *λ* ^ZPE-SRP^ for each transition state to correct the anharmonicity and systematic errors in ZPE calculations for a given model chemistry; for a given model chemistry and a given species this can be factored as

(2) $$\begin{array}{c} \lambda^{\text{ZPE-SRP}}=\lambda^{H}\lambda^{Anh} \end{array}$$

where *λ* ^Anh^ and *λ* ^H^ correct, respectively, the anharmonicity of the ZPE and the inaccuracy of the model chemistry. Note that *λ* ^H^ is characteristic of the model chemistry and *λ* ^Anh^ is characteristic of the species. For all species, we use the harmonic factor *λ* ^H^ that has been parametrized to obtain the accurate harmonic frequency in the F38/10 database [36]. We calculate *λ* ^Anh^ for a given species as the ratio of anharmonic ZPE estimated by hybrid [43], degeneracy-corrected [44], second-order vibrational perturbation theory (HDCVPT2) [45–47] to harmonic ZPE calculated for that species with the same model chemistry. In the calculations of *λ* ^Anh^, the M06-2X/MG3S method was used for the hydrogen abstraction reactions, and the MPW1K/MG3S model chemistry was used for addition reactions. We set *λ* ^ZPE-SRP^ equal to the standard *λ* ^ZPE^ for reactants, pre-reactive van der Waals complexes, and products.

Table 3 lists the calculated *λ* ^ZPE-SRP^ scaling factors of the transition states for all reactions in the present study, as well as the *λ* ^H^ factors and the standard *λ* ^ZPE^ factors. For hydrogen abstraction transition states calculated with M06-2X/MG3S, the table shows that the specific-reaction-parameter scaling factors (*λ* ^ZPE-SRP^ = 0.960–0.966) are smaller than the standard scaling factor (*λ* ^ZPE^ = 0.970). For the addition transition states the calculated *λ* ^ZPE-SRP^ scaling factors are the same as the standard factor *λ* ^ZPE^ (0.983). These results show that the hydrogen abstraction transition states are more anharmonic than the stable reactants or the transition states of the addition reactions.

### 3.2. The SS-QRRK Method

Addition reactions R5-R8 in Figure 1 are treated by the chemical activation mechanism:

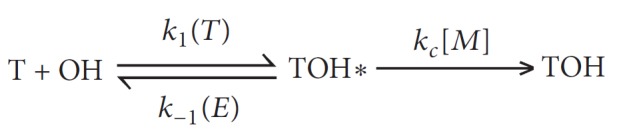
(3)where T denotes the toluene molecule,* T* is temperature, and TOH*∗* is the energized adduct. Applying the steady-state approximation to the energized adduct TOH*∗* yields the phenomenological bimolecular stabilization rate constant,

(4) kstabT1TOHdTOHdt=k1T∑E=E0∞kcMfE,TkcM+k−1E

where the square bracket denotes the concentration, M is the bath gas, and *f*(*E*) is the fraction of energized adduct TOH*∗* with energy* E*. We use the ideal gas law, so [M] equals *p*/*RT*, where* p* is the pressure and* R* is the gas constant.

The three elementary rate constants that need to be determined in (4) are the temperature-dependent high-pressure-limit rate constant *k*_1_(*T*), the energy-dependent dissociation rate constant *k*_−1_(*E*), and collisional deactivation rate constant *k*_*c*_. These are evaluated as follows:

(a) *k*_1_(*T*) is evaluated by the MS-CVT/SCT method explained above.

(b) The high-pressure-limit dissociation rate constants *k*_−1,*∞*_(*T*) is also evaluated by the MS-CVT/SCT method.

(c) We calculate a temperature-dependent Arrhenius preexponential factor *A*_*∞*_(*T*) and Arrhenius activation energy *E*_a_(*T*) by

(5) EaT=−Rdlnk−1,∞Td1/T

(6) A∞T=k−1,∞Texp⁡−EaT/RT

(d) *k*_−1_(*E*) is calculated by using the SS-QRRK method, which is like conventional QRRK theory [48, 49] except that the frequency factor* A* is obtained from (6) and the threshold energy* E*_0_ is obtained from (5). Because *k*_−1,*∞*_(*T*) incorporates variational effects, multidimensional quantum mechanical tunneling, and multistructural anharmonicity, they are also inherited in *k*_−1_(*E*).

(e) *k*_*c*_ is estimated by collision theory as the product of the Lennard-Jones collision rate constant *k*_LJ_ and the collision efficiency *β*_*c*_. The collision efficiency *β*_*c*_ is calculated by

(7) $$\begin{array}{c} \frac{\beta_{c}}{1-\sqrt{\beta_{c}}}=\frac{\left|\langle \Delta E\rangle \right|}{F_{E}k_{B}T} \end{array}$$

where *F*_*E*_ is the energy dependence factor of the density of states calculated by Troe's method [50] and the 〈Δ*E*〉 is the average vibrational energy transferred per collision during both energization and deenergization. In the present study—when not specified otherwise—we set 〈Δ*E*〉 equal to the experimental values [51]: 130 cm^−1^ for Ar and 75 cm^−1^ for He. In the calculations of *k*_LJ_, we based the Lennard-Jones parameters on those used in reference [51] to derive the values of 〈Δ*E*〉; in particular, we used* ε/k*_B_ = 410 K and *σ* = 6.0 Å for the adducts of OH and toluene [52], 120 K and 3.4 Å for Ar [53], and 10 K and 2.55 Å for He [51].

The* Polyrate 2016* [54] and* Gaussrate 17* [55] programs were used for the direct CVT/SCT calculations and SS-QRRK calculations, and the* MSTor* program [56] was used to calculate the conformational-rotational-vibrational partition functions.

### 3.3. Computational Details of Dynamics Calculations

The calculations were converged with respect to the lengths of the calculated reaction paths; in isoinertial coordinates scaled to a mass of 1 amu, the ranges covered were −1.2 to 1.2 Å for addition reactions, −1.5 to 1.5 Å for hydrogen abstraction reactions, and −3.0 to 3.0 Å for all dynamics calculations involving the pre-reactive van der Waals complex of OH with toluene. The step size was set to 0.005 Å for dynamics calculations starting from isolated reactants of all channels except for H-abstraction from* ortho*-site (for which we used a step size of 0.01 Å) and to 0.01 Å for the dynamics calculations starting with the pre-reactive van der Waals complex. For addition reactions we set the ratio of the number of gradients to the number of Hessians to 9 or 10. For the hydrogen abstractions, we calculated more Hessians in order to obtain smooth generalized free energy of activation profiles; in particular we set the* Polyrat*e variable INH equal to 1 or 2 for regions near the saddle point and to 5 for regions away from the saddle point.

## 4. Results and Discussion

### 4.1. Conformers

The reactants, toluene and OH radical, and the products of hydrogen abstraction reactions (R1-R4) are simple molecules that have only one conformer. However, the other species are more complex; there are two torsions, the methyl torsion and the OH torsion, that can produce distinguishable conformers for the transition state of each reaction and the products of the addition reactions (R5-R8). The number of distinguishable conformers that we found for each of the complex species is specified in Table 4. As indicated in (1a), (1b), only the lowest-energy structure of each species is used in the direct dynamics calculations in MS-CVT/SCT calculations; the effect of the other structures on the rate constants is included by the multistructural anharmonicity factor of the reaction, *F*_act_^MS-T^.

**Figure 3:**
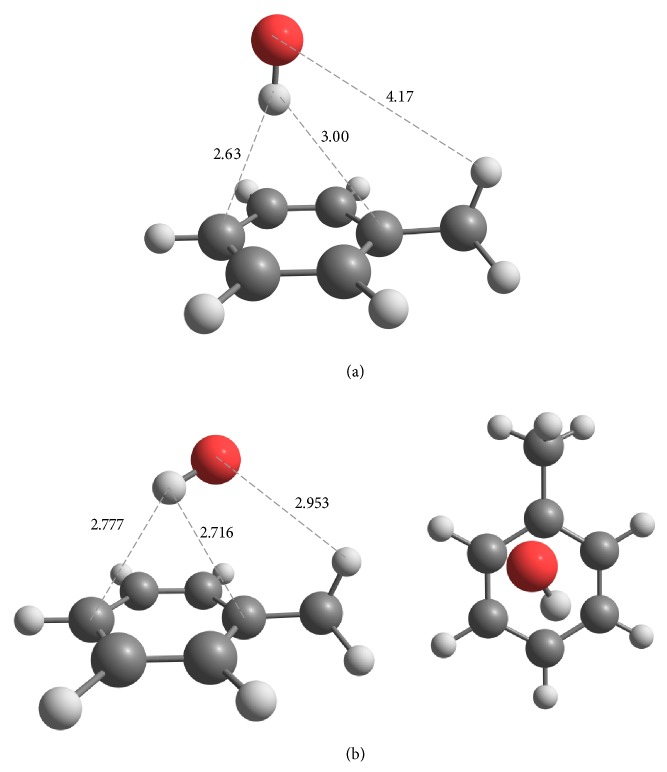
Pre-reactive complex structures: (a) side view of the structure obtained by Uc et al. [10] by the BHandHLYP/6-311++G^*∗∗*^ method, with a *C*_*s*_ symmetry; (b) side and top view of the structure optimized here by the M06-2X/MG3S method. Some bond distances are shown in Å.

We found only one conformer for the pre-reactive complex (see Figure 3(a).) Uc et al. [10] reported a *C*_s_-symmetry structure for the pre-reactive complex by the BHandHLYP/6-311++G^*∗∗*^ method. However, this *C*_s_-symmetry structure was found to be a saddle point with a small imaginary frequency by the M06-2X/MG3S method. We also confirmed that the lowest-energy structures of transition states in all reaction channels share the same pre-reactive complex (Figure 3(b)) by the IRC calculations. Therefore, we used this complex structure in the tunneling calculations for the high-pressure-limit rate constants of the hydrogen abstraction reactions.

### 4.2. Energetics

Tables 5 and 6 give the calculated enthalpies of activation and enthalpies of reaction at 0 K as calculated by using the lowest-energy structures of all species. The tables show that the hydrogen abstraction reaction always has a larger enthalpy of activation than the addition reaction at the same site, and the* ortho*-addition reaction (R5) has the smallest enthalpy of activation. The hydrogen abstraction from methyl group of toluene has the lowest enthalpy of activation among the hydrogen abstraction reactions.

Based on our best estimates, which were obtained by using the CCSD(T)-F12a/jun-cc-pVTZ method for electronic energy and by using the selected model chemistries and the SRP vibrational frequency scaling factors for zero point energy, the calculated enthalpies of activation are in turn -0.16, 3.19, 3.61 and 3.88 kcal/mol for hydrogen abstractions from the methyl group and* ortho*,* meta*, and* para* sites, and they are -0.94, -0.44, 0.26 and 0.56 kcal/mol for addition at* ortho*,* ipso*,* para*, and* meta *sites.

As shown in Table 5, the calculated enthalpy of activation is sensitive to the scaling factor used for vibrational frequencies. Due to the greater anharmonicity of high-frequency modes of transition states of hydrogen abstraction reactions, the enthalpy of activation is overestimated with the standard scaling factor *λ* ^ZPE^ or harmonic scaling factor *λ* ^H^ as compared to that obtained with the SRP scaling factor *λ* ^ZPE-SRP^.

Several groups have investigated the hydrogen abstraction reactions of toluene by OH radical at various theoretical levels. As shown in Table 5, the CCSD(T)-CBS results of Pelucchi et al. [13] are in agreement with the current CCSD(T)-F12a results with standard frequency scaling factors within 0.2 kcal/mol; because they did not account for the greater anharmonicity at the transition states, their enthalpy of activation for abstraction at methyl is 0.8 kcal/mol higher than our best estimate. The G4 method used by Li et al. [12] and the G3 and CBS-QB3 methods used by Seta et al. [8] also overestimate the enthalpy of activation of the hydrogen abstraction reaction from methyl group as compared to our best estimates.

Wu et al. [57] used M06-2X, G3(MP2)-RAD//M06-2X and ROCBS-QB3//M06-2X calculations to calculate the 0 K enthalpies of addition reactions. As shown in Table 6, most of their results overestimate the enthalpy of activation as compared to our best estimates. Their M06-2X and G3(MP2)-RAD calculations predict the same order as we do for the enthalpies of activation for addition reactions, with the* ortho*-site preferred. However, their ROCBS-QB3 results give a different order and predict the* meta* addition to have the lowest enthalpy of activation at 0 K.

### 4.3. Multistructural Anharmonicity

**Figure 4:**
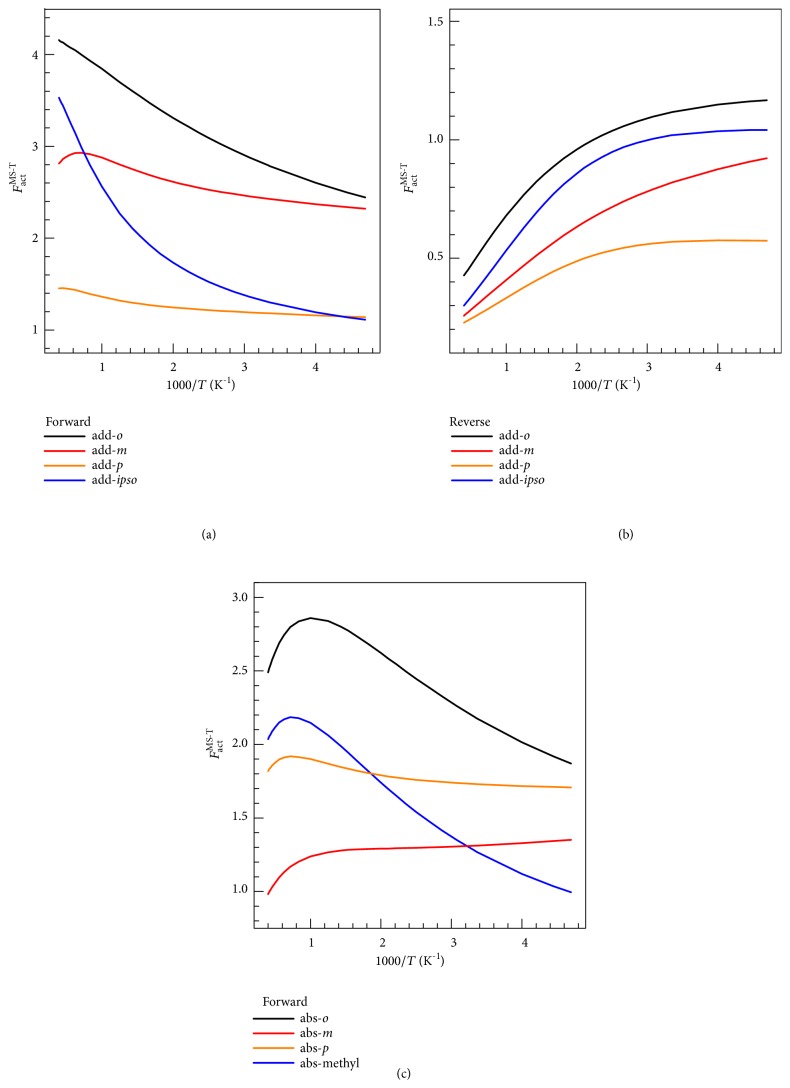
Multistructural anharmonicity factors *F*_act_^MS-T^ for (a) the forward addition reaction rates, (b) their reverse reaction rates, and (c) the forward abstraction reaction rates calculated by the MS-T method.

We included the effect of multistructural anharmonicity due to molecular torsions by the multistructural torsion (MS-T) method, which corrects the rate constants obtained in the single-structure quasiharmonic approximation by multiplying by the multistructural anharmonic factor of the reaction. In the MS-T calculations, the coupled torsional potential is used for the transition states of* ipso* addition and* ortho* and methyl abstraction and for the products of* ipso* and* ortho*-addition because in these cases, the torsions that introduce the multistructural torsional anharmonicity are coupled with each other because of steric effects. For the other complex species, the uncoupled torsional potential is used because the torsions considered are only weakly coupled with each other. The calculated multistructural anharmonic factors *F*_act_^MS-T^ as functions of temperature are shown in Figure 4. The figure shows that the effect of multistructural anharmonicity usually increases with temperature, and it can change the calculated reaction rates by factors as small as 0.2 and as large as 4.2.

### 4.4. Transmission Coefficients

**Figure 5:**
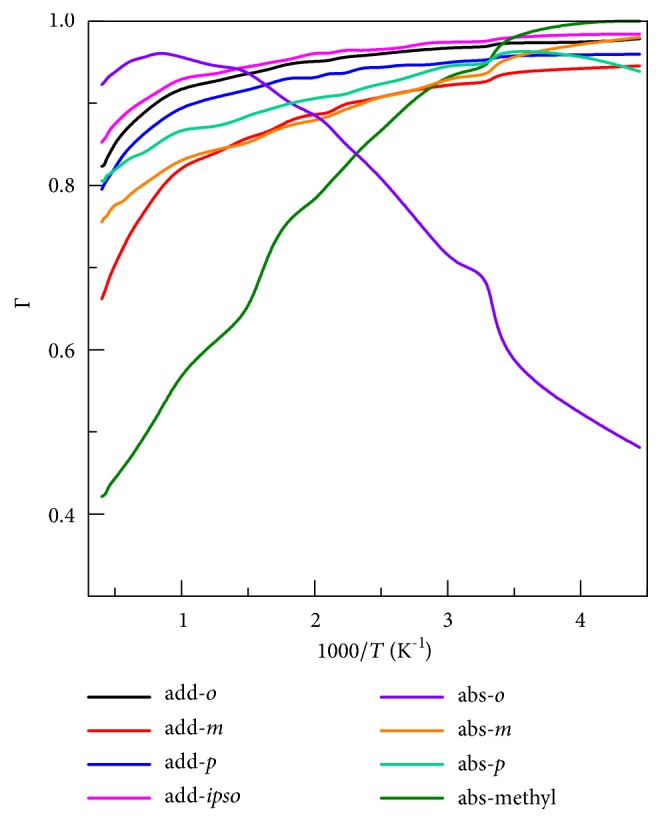
The variational effect (Γ = *k*_CVT_/*k*_*TST*_).

**Figure 6:**
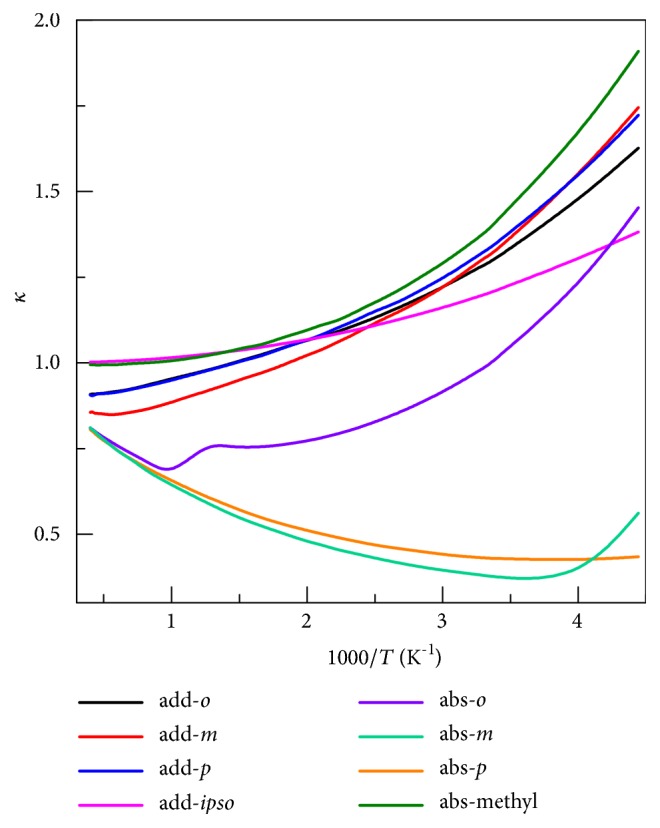
The tunneling transmission coefficient (*κ* ^*SCT*^) calculated in the high-pressure limit with the small-curvature tunneling approximation.

The recrossing transmission coefficient Γ and the tunneling transmission coefficient *κ* ^*SCT*^ are important components of the dynamics calculations, and they are shown for each reaction (in the high-pressure limit) in Figures 5 and 6, respectively.

Figure 5 shows that the most important recrossing coefficients are for H-abstraction from methyl and from the* ortho* site. These recrossing transmission coefficients can decrease the rate constants by more than a factor of two.

Figure 6 shows that the tunneling transmission coefficient ranges from 0.35 to 1.9, where a value less than unity corresponds to nonclassical reflection dominating tunneling in the thermal average, and a value greater than unity corresponds to tunneling dominating nonclassical reflection in the thermal average. At 275 K, the tunneling transmission coefficient increases the ratio of abstraction from methyl to abstraction from the* para* position by a factor of 4.4, and the effect is even larger at lower temperatures.

### 4.5. High-Pressure-Limit (HPL) Rate Constants

**Figure 7:**
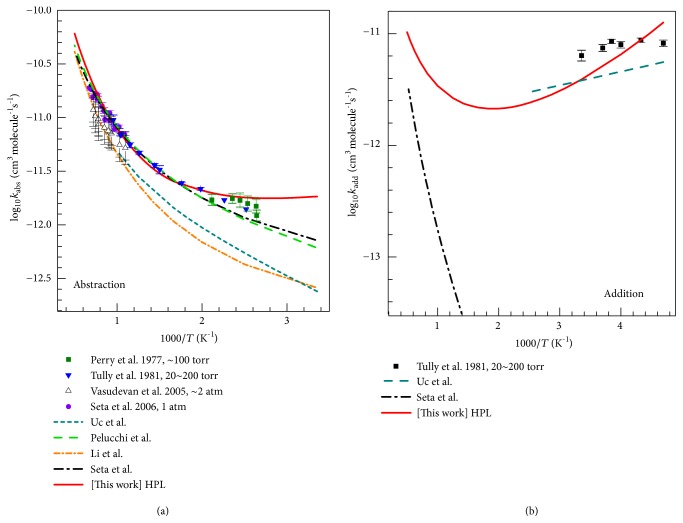
The reaction rate constants in the high-pressure limit: (a) sum of rate constants for abstraction reactions; (b) sum of rate constants for addition reactions. The SRP scaling factor is used for vibrational frequencies of transition states. For all reactions, tunneling is allowed to occur at all energies above the ZPE of the pre-reactive van der Waals complex. Some experimental data (symbols) and previous theoretical results (lines) are also given for comparison.

Figures 7(a) and 7(b) show the temperature-dependent HPL rate constants calculated by the MS-CVT/SCT method for the hydrogen abstraction reactions and addition reactions, respectively, and for comparison experimental data is also displayed in the figure. The hydrogen abstraction reactions dominate at high temperatures, whereas the addition reactions dominate at low temperatures. Therefore, we plot the experiment data at 213 K–298 K and 504 K–1481 K as rate constants of the addition reactions and the abstraction reactions, respectively.

#### 4.5.1. High-Pressure-Limit (HPL) Rate Constants: Abstraction

Figure 7(a) shows that our total abstraction rate constants agree well with the experimental results of Seta et al. [8] and Tully et al. [7] for the mid-temperature range (500 K <* T* < 1000 K) and are slightly higher (by a factor of 1.4) than the experimental data in the temperature interval 1000–1500 K. The calculations of Li et al. [12] and Uc et al. [10] are in good agreement with each other, but their results are lower than our results and those estimated by Pelucchi et al. [13] because both Li et al. and Uc et al. overestimated the barrier height for hydrogen abstraction from the methyl group as we saw in Table 5.

The comparison of our abstraction rate constants with the work of Pelucchi et al. is particularly interesting because their work is in some respects similar to ours. They used conventional transition state theory with a correction for variational-transition-state-location effects computed by M06-2X/6-311+G(d,p). They computed the classical barrier height by CCSD(T)/aug-cc-pVTZ//M06-2X/6-311+G(d,p) with a correction for basis set effects, and they calculated frequencies by M06-2X/6-311+G(d,p) with no mention of frequency scaling. (The 6-311+G(d,p) basis set they used is similar to the MG3S basis set used here for direct dynamics because for systems containing only C, H, and O, the MG3S basis set is equivalent to 6-311+G(2df,2p), which has more polarization functions than 6-311+G(d,p).) They included torsional anharmonicity by one-dimensional and two-dimensional models applied only at the transition state. They used an asymmetric Eckart model to evaluate the tunneling transmission coefficient. (The Eckart model is less reliable than the SCT method employed here because it does not include the true shape of the effective potential energy barrier, and it neglects corner-cutting tunneling.) The net result of their calculation is in good agreement with our results (apparently due to some cancellation among the various factors that differ) at most temperatures, but there is a significant deviation for* T* < 450 K.

**Figure 8:**
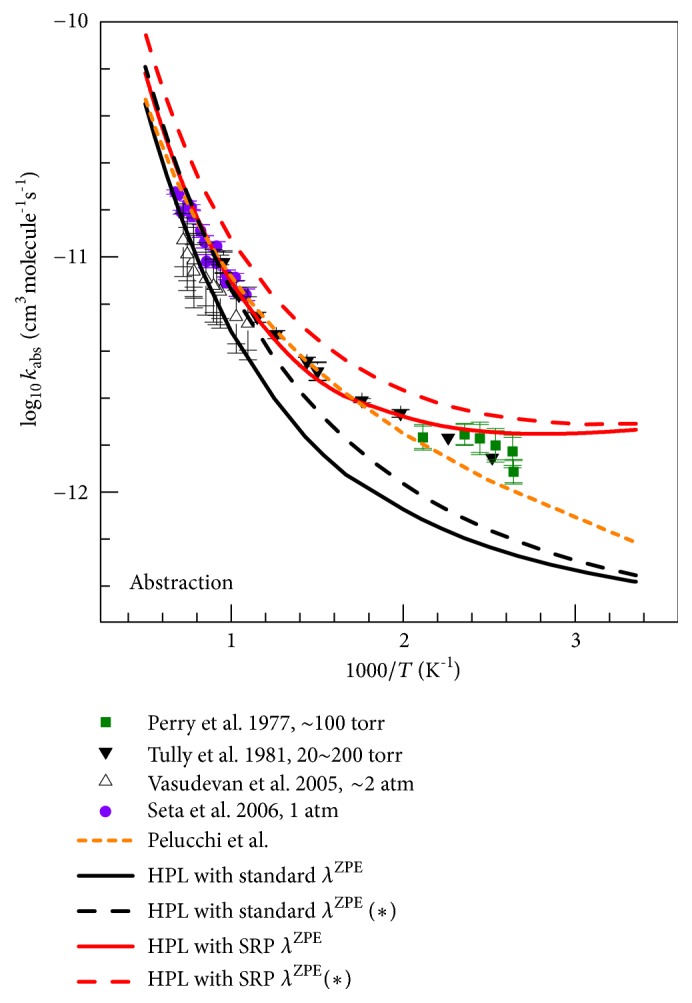
Effects of scaling factors and variational effects on the reaction rate constants of abstraction reactions; the curves labeled with asterisks in parentheses are obtained by conventional transition state theory (no variational-transition-state-location effects).

Figure 8 shows the effects of frequency scaling factors. The figure clearly shows that using the standard scaling factors *λ* ^ZPE^ greatly underestimates the rate constants; for example, the HPL rate constant calculated with the standard *λ* ^ZPE^ factors is lower than that obtained with the SRP factor *λ* ^ZPE-SRP^ by a factor of 0.72 at 1600 K, by a factor of 0.40 at 500 K, and by a factor of 0.13 at 213 K.

Figure 8 also shows the variational-transition-state-location effects. We find a variational-transition-state-location effect increasing with temperature up to about 30% (i.e., decreasing the calculated rate constant by about 30%) at high* T*; this is in good agreement with the calculations of Pelucchi et al.

The most important conclusion from Figure 8 is that if we neglect variational-transition-state-location effects or do not account for the transition state anharmonicity being greater than predicted by the standard model, the agreement with experiment is noticeably degraded.

#### 4.5.2. High-Pressure-Limit (HPL) Rate Constants: Addition

The total HPL rate constants of addition reactions are plotted in Figure 7(b), and they agree reasonably with the experimental data of Tully et al. at* T *= 213–298 K.

### 4.6. Pressure-Dependent Rate Constants

**Figure 9:**
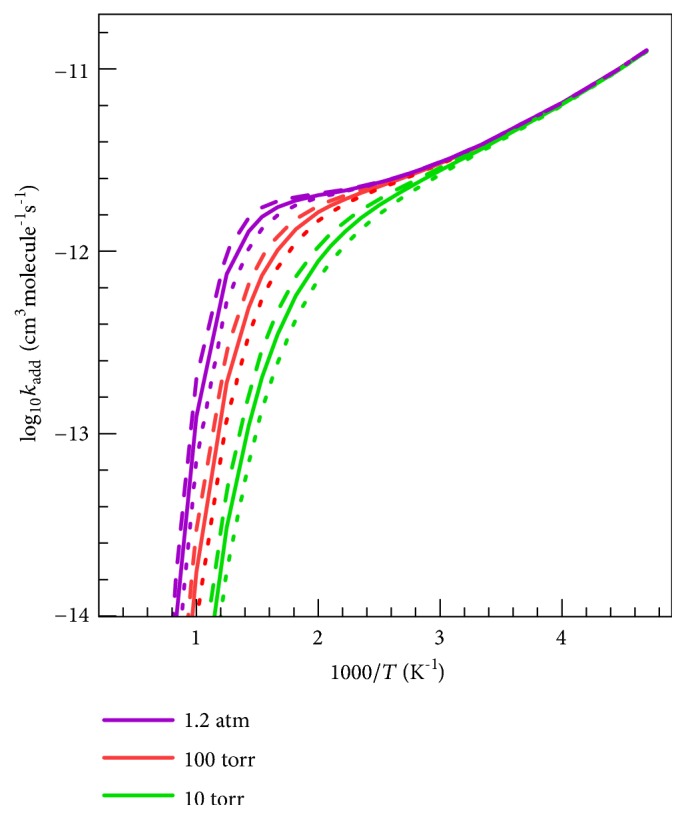
Rate constants of addition reactions at various pressures calculated by the SS-QRRK method with Ar as bath gas. The figure also presents the Δ*E* sensitivity: Δ*E* = 65 cm^−1^ (the dot line), Δ*E* = 130 cm^−1^ (the solid line), and Δ*E* = 260 cm^−1^ (the dash line).

Figure 9 illustrates the calculated pressure dependence of the total rate constant for addition reaction with Ar as the bath gas. We see from (7) that the collision efficiency increases with increasing 〈Δ*E*〉, and consequently, the collision rate constant *k*_*c*_ increases with increasing 〈Δ*E*〉. The addition rate constant therefore increases with increasing 〈Δ*E*〉 because the adducts are more readily stabilized. These expectations are consistent with the values in the figure, where the solid curves are for the experimental value of 〈Δ*E*〉, namely 130 cm^−1^, and we also show what the results would be if 〈Δ*E*〉 were a factor-of-two larger or a factor-of-two smaller. The effect of varying 〈Δ*E*〉 is relatively small; for example, at* p *= 100 Torr and* T* = 500 K, when 〈Δ*E*〉 is halved to 65 cm^−1^, the rate constant is decreased by a factor of 0.90 as compared to that obtained by using 〈Δ*E*〉 = 130 cm^−1^; and using two times the 〈Δ*E*〉 value, that is 260 cm^−1^, the rate constant is increased by a factor of 1.08.

We see that the pressure dependence sets to an appreciable extent for* T *>  ~330 K, and the fall-off becomes steeper at temperatures higher than 500 K. The high-temperature results in Figure 9 look nothing like the high-temperature results in Figure 7(b) because 1.2 atm (the highest pressure in Figure 9) is far from the high-pressure limit at high* T*. However, at low* T*, all the curves in Figure 9 are at the high-pressure limit.

**Figure 10:**
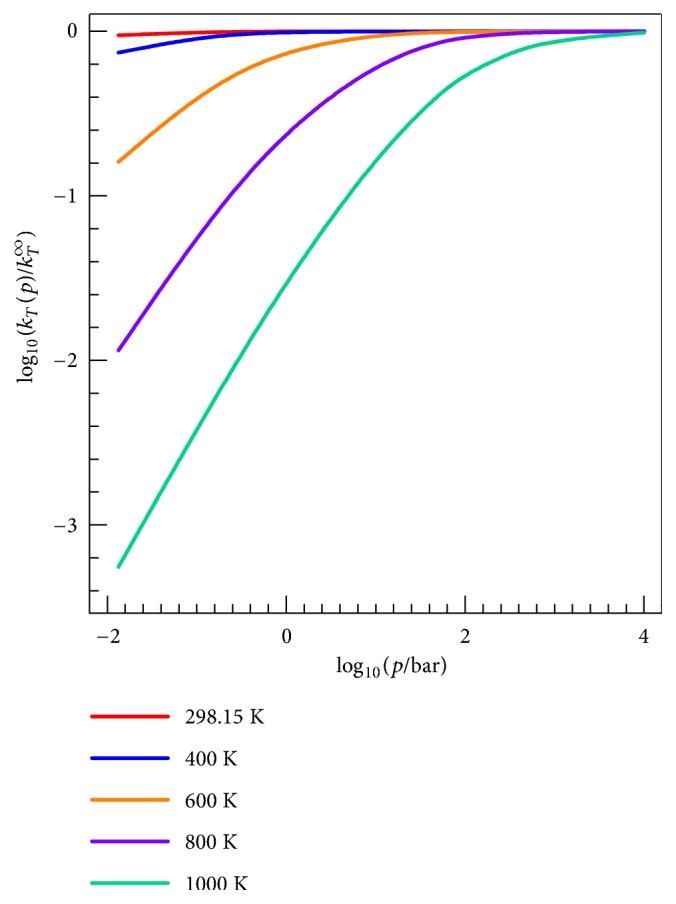
Fall-off curves of addition reactions computed by the SS-QRRK method with Ar as bath gas. The HPL addition rate constant is *k*_*T*_^*∞*^.

Figure 10 illustrates the calculated pressure dependence of the total rate constant for addition reaction over a wider pressure range; this figure is for Ar as the bath gas, and it shows results only for the experimental value of 〈Δ*E*〉. Figure 10 shows that at* T* = 298.15 K, there is almost no pressure dependence for pressures larger than 100 Torr (log_10_ *p*/bar = –0.9), and this result is in agreement with experimental observations [7]. The experiments of Tully in Figure 7(b) are for* T* ≤ 298 K and for log_10_ *p*/bar in the range -1.6 to -0.6. Figure 10 shows that this is in the high-pressure limit, so the comparison of HPL rate constants to experiment in Figure 7(b) is warranted.

### 4.7. Total Rate Constants

**Figure 11:**
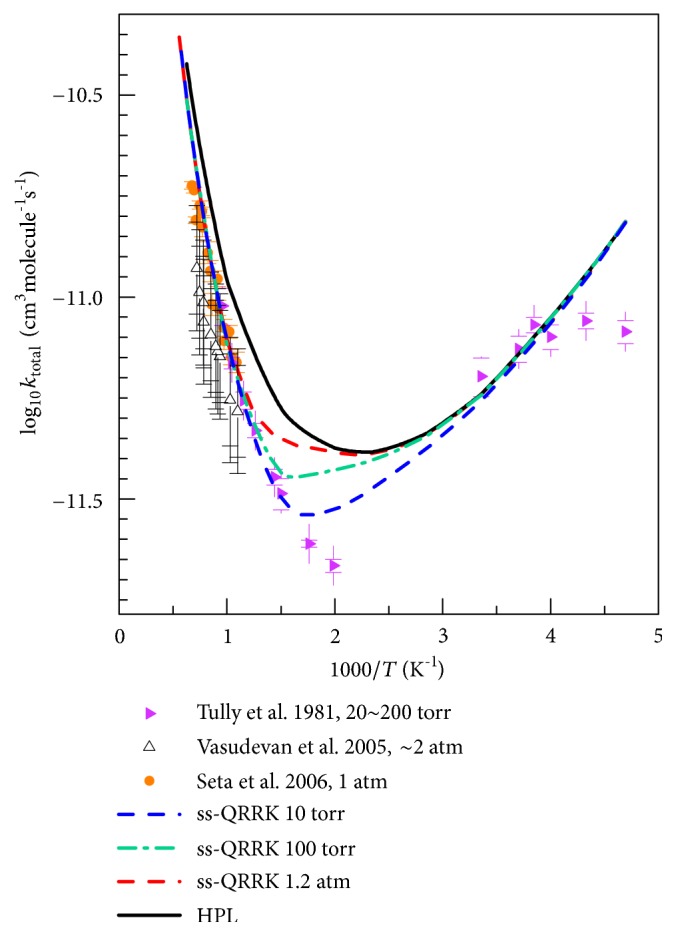
Total rate constants (sum of rate constants of all addition and abstraction reactions). The curves are the present theoretical results by MS-CVT/SCT with fall-off effects treated by SS-QRRK with Ar as bath gas; HPL denotes the high-pressure limit. The symbols are experimental results.

Figure 11 shows the final total reaction rate constants calculated as the sum of the rate constants of R1-R8 reactions at several pressures, and it compares the calculations to the available reliable experimental data; Table 7 lists a few specific values.

In the high-temperature region (*T* > 1000 K), the calculated total reaction rate constant at the experimental pressures (20 Torr to ~1 atm) is dominated by the hydrogen abstraction reaction (which is pressure-independent in our PEM model), and the current calculations agree well with the available experiments. As given in Table 7, we overestimate the rate constants at 500 K and 568 K by a factor of ~1.5–1.7 as compared to those obtained by Tully in Ar, but only slightly underestimate the rate constants at 298.15 K. At 230–300 K, we reproduce the experimental data very well. At very low* T*, below 230 K, we overestimate the experimental results by as much as a factor of 2.5.

In the mid-temperature range (700–350 K), our calculations reveal the nonmonotonic temperature dependence and the strong pressure dependence of the total rate constants (at 500 K and 10 Torr, the rate constant is calculated to be 31% lower than the HPL). The nonmonotonic temperature dependence is a result of competition of the addition reactions and hydrogen abstraction reactions. The experimental results are very uncertain in this region.

### 4.8. Branching Fractions

**Figure 12:**
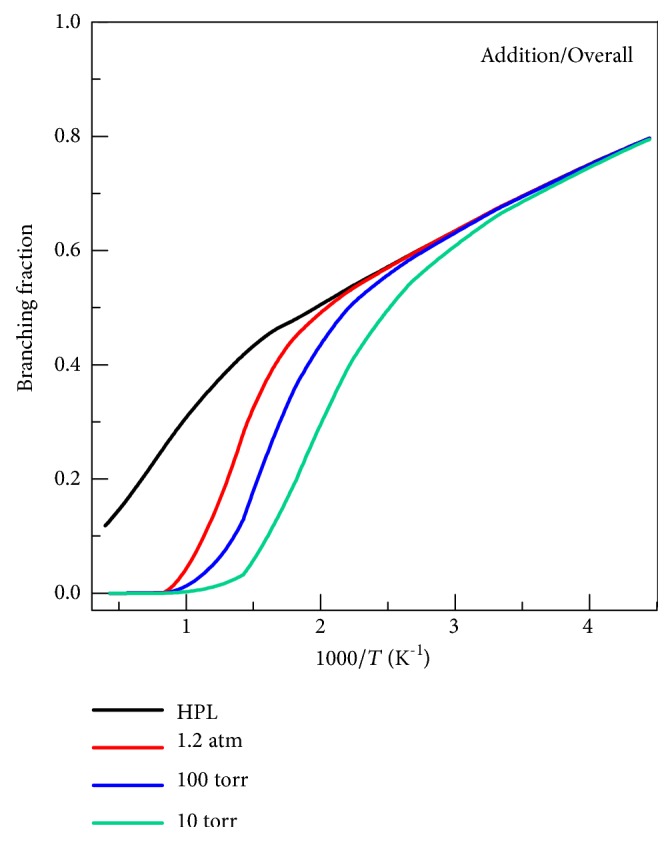
The total branching fraction of the addition reactions in Ar as a function of temperature.

An advantage of theoretical studies is that they can yield rate constants for individual products, whereas experimental studies are usually based on loss of reactant and so yield only total rate constants.  Figure 12 shows the total addition fraction at various pressures in Ar, where this branching fraction is defined as the sum of the addition rate constants divided by the sum of all the rate constants (abstraction and addition). In the low-temperature region (213 K <* T* < 300 K) that is important for atmospheric chemistry, the reaction is mainly dominated by the addition reactions with a total branching fraction larger than ~0.7.  Figure 12 also shows that in the HPL the abstraction reaction dominates for* T* > 500 K. At lower pressure the switch in dominance occurs at lower temperatures, for example for 10 Torr it occurs at 400 K.

**Figure 13:**
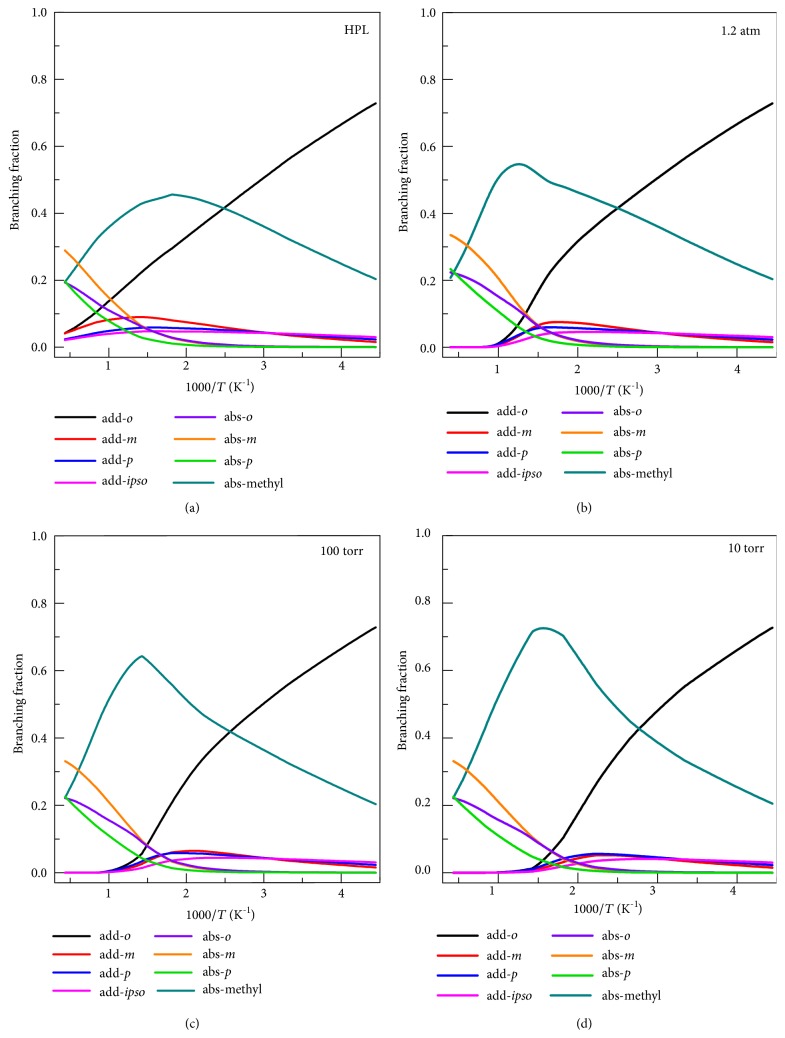
The branching fraction of each reaction in the high-pressure limit (HPL), 1.2 atm, 100 Torr, and 10 Torr (with Ar as bath gas) as a function of temperature.

Figure 13(a) shows HPL branching fractions for each of the eight reactions. It shows that the hydrogen abstraction reactions from the methyl group and meta site are the dominant reactions at high temperatures, and at low temperatures, the* ortho*-addition reaction has a significant dominance. We also notice the significant contribution of hydrogen abstraction from the methyl at low* T*. This is explained by the obtained negative (near-zero) enthalpy of activation at 0 K when the anharmonicity of the high-frequency modes of transition state is considered by using a SRP scaling factor of frequency, as shown in Table 5. At 298 K, we predict the contribution of methyl hydrogen abstraction to be ~30%, which is larger than the branching ratio (7%) used in some atmospheric chemistry models [57], for example, MCM3.2 and SAPRC-11. The present results could be used to update those models.

Figures 13(b)–13(d) show branching fractions at* p* = 1.2 atm, 100 Torr, and 10 Torr with Ar as bath gas. We observe significant pressure effects on the fractions of the two dominant reactions, addition at the* ortho*-site and abstraction from the methyl site. The main effect of decreasing the pressure from the HPL to 1.2 atm is that the addition reaction essentially turns off above 1000 K, and abstraction from the methyl increases significantly for temperatures above 600 K. The peak of the branching fraction curve for abstraction from the methyl site increases and moves to higher temperature; the maximum increases from 0.45 at ~550 K (in HPL) to 0.78 at ~650 K (at* p* = 10 Torr). With the decrease of pressure, the temperature where the dominant reaction shifts from* ortho*-addition and abstraction at methyl switches shifts from ~400 K in HPL to 325 K at 10 Torr. At 10 Torr, the addition reactions have essentially turned off by 700 K.

## 5. Summary

In this work, we present a theoretical and computational study of the kinetics of toluene reaction with OH over a wide temperature range from 213 K to 2500 K. We focus on hydrogen abstraction and addition reaction channels (R1-R8), which dominate, respectively, at high and low temperatures. The high-pressure limit rate constants of all channels have been estimated with multistructural canonical variational transition state theory with the small-curvature tunneling approximation (MS-CVT/SCT) by using the multistructural torsional anharmonicity (MS-T) method to take account of anharmonicity resulting from torsional motions and multiple conformers. We also corrected the anharmonicity of high-frequency modes of the transition states by using the specific-reaction-parameter (SRP) scaling factors for vibrational frequencies.

The pressure-dependent rate constants of addition reactions at several pressures have been estimated by the SS-QRRK method. The final total reaction rate constants calculated as a sum of the rate constants of reactions R1–R8 are compared to the available experimental data. We get good agreement with those experimental results in the temperature region in which the experiments appear to be most reliable. We predict the very weak pressure dependence of the rate constants for* T* < 300 K, but obtain strongly pressure-dependent reaction rates at higher temperatures. We also observe significant contributions of hydrogen abstraction from the methyl group even at low temperature (it accounts for ~30% and ~20% of the total rate at 298 K and 213 K, respectively), and that could be important for understanding the fate of alkyl aromatics in the atmosphere.

## Figures and Tables

**Table 1:** *T*_1_ diagnostics^*a*^.

Species	*T*_1_	Species^*a*^	*T*_1_
C_6_H_5_OH	0.011	OH	0.008
*o*-C_6_H_4_CH_3_	0.013	TS-abs-*o*	0.023
*m*-C_6_H_4_CH_3_	0.014	TS-abs-*m*	0.023
*p*-C_6_H_4_CH_3_	0.013	TS-abs-*p*	0.023
C_6_H_5_CH_2_	0.020	TS-abs-CH_3_	0.022
*o*-C_6_H_5_OHCH_3_	0.016	TS-add-*o*	0.028
*m*-C_6_H_5_OHCH_3_	0.017	TS-add-*m*	0.028
*p*-C_6_H_5_OHCH_3_	0.016	TS-add-*p*	0.028
*ipso*-C_6_H_5_OHCH_3_	0.016	TS-add-*ipso*	0.029
H_2_O	0.009		

^*a*^TS denotes transition structure.

**Table 2:** Classical energies of reaction and barrier heights (kcal/mol)^*a*^.

Channel	CCSD(T)-F12a^*b*^			M06-2X/MG3S			M08-SO/MG3S			MN15/MG3S		
	*V*_*f*_^‡^,	*V*_*r*_^‡^,	∆*E*	*V*_*f*_^‡^,	*V*_*r*_^‡^,	∆*E*	*V*_*f*_^‡^,	*V*_*r*_^‡^,	∆*E*	*V*_*f*_^‡^,	*V*_*r*_^‡^,	∆*E*
*o-*abs (R1)	5.57	11.80	-6.23	4.80	11.11	-6.31	4.99	10.20	-5.21	4.78	11.78	-7.00
*m*-abs (R2)	6.08	12.20	-6.12	5.44	11.72	-6.28	5.69	10.79	-5.10	5.45	12.39	-6.94
*p-*abs (R3)	6.29	11.94	-5.65	5.64	11.43	-5.79	5.93	10.49	-4.56	5.66	12.08	-6.42
CH_3_-abs (R4)	1.65	29.89	-28.24	1.33	28.23	-26.90	0.90	28.83	-27.93	0.25	29.78	-29.53
MUD^*c*^	0.00			0.62			0.92			0.63		

*o*-add (R5)	-2.51	19.16	-21.67	-1.51	20.36	-21.87	-3.27	19.53	-22.80	-4.22	19.88	-24.10
*m-*add (R6)	-0.84	19.53	-20.37	0.66	20.86	-20.20	-1.03	20.11	-21.14	-2.02	20.28	-22.30
*p*-add (R7)	-1.23	19.32	-20.55	0.05	20.69	-20.64	-1.68	20.03	-21.71	-2.62	20.17	-22.79
*ipso*-add (R8)	-1.93	20.24	-22.17	-0.51	21.11	-21.62	-2.36	19.95	-22.31	-3.35	20.42	-23.77
MUD^*c*^	0.00			0.92			0.58			1.37		

^*a*^ *V*_*f*_^‡^, *V*_*r*_^‡^, and ∆*E* are the forward classical barrier height, the reverse classical barrier height, and the classical energy of reaction, respectively.

^*b*^The basis set is jun-cc-pVTZ, and the geometries are optimized by the M08-HX/MG3S method.

^*c*^MUD is the mean unsigned deviation from the best estimates: *MUD* = Σ_*j*_^12^|*E*_*j*_^*KS*^ − *E*_*j*_^*CCSD*(*T*)^|/12, where *j *goes through all barrier heights and reaction energies for the abstraction (top half of table) or addition (bottom half of table) reactions.

**Table 3:** Scaling factors for transition state frequencies.

	abstraction reactions^*a*^	all addition reactions
*λ* ^H^	0.982	0.995^*b*^
Standard *λ* ^ZPE^	0.970	0.983^*b*^
*λ* ^ZPE-SRP^	*o*-abs: 0.964	0.983^*c*^
	*m*-abs: 0.965	
	*p*-abs: 0.966	
	CH_3_-abs: 0.960	

^*a*^Calculated by M06-2X/MG3S.

^*b*^Calculated by M08-SO/MG3S.

^*c*^As explained in in the text, these factors are the product of *λ* ^H^ calculated by M08-SO/MG3S and *λ* ^Anh^ calculated by MPW1K/MG3S.

**Table 4:** Conformers and torsion schemes.

species	number of conformers^*a*^	scheme^*b*^
TS_add-*o*_	2 (1)	U
TS_add-*m*_	2 (1)	U
TS_add-*p*_	1	U
TS_add-*ipso*_	1	C
P_add-*o*_	4 (2)	C
P_add-*m*_	4 (2)	U
P_add-*p*_	6 (3)	U
P_add-*ipso*_	3 (1)	C
TS_abs-*o*_	2 (1)	C
TS_abs-*m*_	2 (1)	U
TS_abs-*p*_	2	U
TS_abs-CH_3__	2	C

^*a*^The number in parentheses represents how many pairs of mirror structures are included in the conformers.

^*b*^U denotes that the two defined torsions are considered as uncoupled, and C denotes that they are treated as coupled.

**Table 5:** Enthalpies of activation and reaction at 0 K (in kcal/mol) for hydrogen abstraction reactions.

	This work						Pelucchi et al.	Li et al.	Seta et al.	
	M06-2X/MG3S			CCSD(T)-F12/jun-cc-pVTZ^*a*^			CCSD(T)/CBS^*b*^	G4	G3(MP2)	CBS-QB3
	with *λ* ^H^	with standard *λ* ^ZPE^	with SRP *λ* ^ZPE^	with *λ* ^H^	with standard *λ* ^ZPE^	with SRP *λ* ^ZPE^				
Enthalpy of activation										
*o*-abs (R1)	2.91	2.93	2.42	3.68	3.70	3.19	3.5	3.2	6.07	3.01
*m*-abs (R2)	3.35	3.38	2.96	4.00	4.03	3.61	3.8	3.5	6.64	3.70
*p*-abs (R3)	3.55	3.58	3.24	4.20	4.22	3.88	4.0	3.6	7.10	3.32
CH_3_-abs (R4)	0.35	0.36	-0.49	0.68	0.69	-0.16	0.6	1.2	2.77	1.24
Enthalpy of reaction										
*o*-abs (R1)	-6.27	-6.27	-6.27	-6.19	-6.19	-6.19	-6.1	-6.2	-2.94	-4.82
*m*-abs (R2)	-6.32	-6.32	-6.32	-6.16	-6.16	-6.16	-6.2	-6.1	-2.94	-4.88
*p*-abs (R3)	-5.84	-5.84	-5.84	-5.63	-5.69	-5.69	-5.7	-5.6	-2.34	-3.70
CH_3_-abs (R4)	-27.29	-27.12	-27.12	-28.63	-28.46	-28.46	-28.3	-28.5	-25.60	-28.47

^*a*^The ZPEs are obtained by M06-2X/MG3S with scaled frequencies.

^*b*^Electronic energies are calculated as *E*_CCSD(T)/aug-cc-pVTZ_ + *E*_DF-MP2/aug-cc-pVQZ_ − *E*_DF-MP2/aug-cc-pVTZ_ with the M06-2X/6-311+G(d,p) geometries, and the ZPEs are obtained by M06-2X/6-311+G(d,p).

**Table 6:** Enthalpies of activation and reaction at 0 K (in kcal/mol) for addition reactions.

	This work				Wu et al.		
	M08-SO/MG3S		CCSD(T)-F12/ jun-cc-pVTZ^*a*^		M06-2X^*b*^	G3(MP2)-RAD^*c*^	CBS^*d*^
	with *λ* ^H^	with *λ* ^ZPE^	with *λ* ^H^	with *λ* ^ZPE^			
Enthalpy of activation							
*o*-add (R5)	-1.68	-1.70	-0.92	-0.94	0.14	0.50	1.46
*m-*add (R6)	0.38	0.37	0.57	0.56	2.22	2.22	-0.05
*p*-add (R7)	-0.17	-0.19	0.28	0.26	1.41	1.53	0.12
*ipso*-add (R8)	-0.86	-0.87	-0.43	-0.44	1.00	0.86	1.48
Enthalpy of reaction							
*o*-add (R5)	-19.60	-19.64	-18.48	-18.52	-18.74	-17.66	-19.00
*m-*add (R6)	-18.05	-18.09	-17.28	-17.32	-17.04	-16.40	-17.50
*p*-add (R7)	-18.60	-18.64	-17.44	-17.48	-17.38	-16.37	-17.73
*ipso*-add (R8)	-19.26	-19.30	-19.12	-19.16	-18.48	-18.48	-19.62

^*a*^The ZPEs are obtained by M08-SO/MG3S with scaled frequencies.

^*b*^M06-2X/6-311++G(2df,2p).

^*c*^G3(MP2)-RAD//M06-2X/6-311++G(2df,2p). The meaning of “//” is that geometries and frequencies recalculated by the method after the double slash, and single-point energies are calculated by the method before the double slash.

^*d*^ROCBS-QB3//M06-2X/6-311++G(2df,2p).

**Table 7:** The total rate constants (10^12^ cm^3^ molecule^−1^ s^−1^) as functions of temperature and pressure.

*T* (K)	*P* (Torr)	present	Davis^*a*^	Hensen^*b*^	Perry^*c*^	Tully^*d*^
298.15	100 (He)	5.69				6.0±0.4
298.15	40 (He)	3.82				5.4±0.1
298.15	20 (He)	3.77	5.1±0.2			4.7±0.5
298.15	3 (He)	3.37	3.6±0.3			
298.15	100 (Ar)	5.75		5.8±0.6	6.4±0.6	6.4±0.7
500	100 (Ar)	3.72				2.2±0.1^*e*^
500	10 (Ar)	2.95				
568	100 (Ar)	3.63				2.4±0.1^*e*^
568	10 (Ar)	2.88				

^*a*^Reference [3]. ^*b*^Reference [5]. ^*c*^Reference [6]. ^*d*^Reference [7]. ^*e*^At a pressure of 20-200 Torr.

## Supplementary Materials

Supplementary Materials
* Table S1*: fitting parameters for the high-pressure-limit rate constants of the forward reactions of R1-R8.* Table S2*: fitting parameters for the high-pressure-limit rate constants (k–1) of the reverse reactions of R5-R8.* Table S3*: the Arrhenius pre-exponential factor *A*_*∞*_(*T*) (s^−1^) and Arrhenius activation energy *E*_*a*_(*T*) (kcal/mol) of the high-pressure-limit rate constants of reverse reactions of R5-R8. These are obtained using the fitting parameters of* k*_–1_.* Table S4*: energy dependence factor of the density of states (*F*_*E*_) as a function of temperature as calculated by the Whitten-Rabinovitch method.* Table S5*: collision efficiency *β*_*c*_ and collisional deactivation rate constant *k*_*c*_ (cm^3^molecule^−1^s^−1^) as functions of temperature.
