# Supplementary material for: Kinetics of the Toluene Reaction with OH Radical
Source: Research (Wash D C). 2019 May 29;2019:5373785. doi: 10.34133/2019/5373785 (PMC6750082; doi:10.34133/2019/5373785)
Supplement: Supplementary Materials — Table S1: fitting parameters for the high-pressure-limit rate constants of the forward reactions of R1-R8. Table S2: fitting parameters for the high-pressure-limit rate constants (k–1) of the reverse reactions of R5-R8. Table S3: the Arrhenius pre-exponential factor A ∞(T) (s−1) and Arrhenius activation energy E a(T) (kcal/mol) of the high-pressure-limit rate constants of reverse reactions of R5-R8. These are obtained using the fitting parameters of k –1. Table S4: energy dependence factor of the density of states (F E) as a function of temperature as calculated by the Whitten-Rabinovitch method. Table S5: collision efficiency β c and collisional deactivation rate constant k c (cm3molecule−1s−1) as functions of temperature. [file 5373785.f1.zip › 5373785.f1/5373785.f1.docx]

**Table S1 Fitting parameters for the high-pressure-limit rate constants of the forward reactions of R1-R8.Table S2 Fitting parameters for the high-pressure-limit rate constants (k–1) of the reverse reactions of R5-R8.**

**Table S3** The Arrhenius pre-exponential factor $A_{\infty}\left( T \right)$ (s^-1^) and Arrhenius activation energy $E_{a}\left( T \right)$ (kcal/mol) of the high-pressure-limit rate constants of reverse reactions of R5-R8. These are obtained using the fitting parameters of *k*_–1_.

**Table S4** Energy dependence factor of the density of states ${(F}_{E})$ as a function of temperature as calculated by the Whitten-Rabinovitch method

**Table S5** Collision efficiency $\beta_{c}$ and collisional deactivation rate constant $k_{c}$ (cm^3^ molecule^-1^ s^-1^) as functions of temperature
