# Supplementary material for: Kinetics of the Toluene Reaction with OH Radical
Source: Research (Wash D C). 2019 May 29;2019:5373785. doi: 10.34133/2019/5373785 (PMC6750082; doi:10.34133/2019/5373785)
Supplement: Supplementary Materials — Table S1: fitting parameters for the high-pressure-limit rate constants of the forward reactions of R1-R8. Table S2: fitting parameters for the high-pressure-limit rate constants (k–1) of the reverse reactions of R5-R8. Table S3: the Arrhenius pre-exponential factor A ∞(T) (s−1) and Arrhenius activation energy E a(T) (kcal/mol) of the high-pressure-limit rate constants of reverse reactions of R5-R8. These are obtained using the fitting parameters of k –1. Table S4: energy dependence factor of the density of states (F E) as a function of temperature as calculated by the Whitten-Rabinovitch method. Table S5: collision efficiency β c and collisional deactivation rate constant k c (cm3molecule−1s−1) as functions of temperature. [file 5373785.f1.zip › 5373785.f1/5373785.f2.docx]

**Supplementary Information**

**Table S1** Fitting parameters for the high-pressure-limit rate constants of the forward reactions of R1-R8

| Path | R1 | R2 | R3 | R4 | R5 | R6 | R7 | R8 | Total |
| --- | --- | --- | --- | --- | --- | --- | --- | --- | --- |
|  | *o*-abs | *m*-abs | *p*-abs | methyl-abs | *o*-add | *m*-add | *p*-add | *ipso*-add |  |
| $\ln A$ | -13.09 | -13.11 | -13.47 | -13.15 | -13.66 | -13.39 | -13.81 | -14.18 | -13.7 |
| $n$ | 2.889 | 3.168 | 3.443 | 2.578 | 2.289 | 2.197 | 2.327 | 2.651 | 3.854 |
| $E$ | 1.493 | 1.715 | 2.455 | -2.206 | -5.299 | -1.362 | -2.003 | -3.152 | -6.085 |
| $T_{0}$ | -136 | -144.8 | -133.2 | 110.1 | 4.025 | 30.46 | 67.93 | 36.9 | 1.023 |

Fitted by using $k=A\left( \frac{T+T_{0}}{300} \right)^{n}e^{-\frac{E(T+T_{0})}{R(T^{2}+T_{0}^{2})}}$; the units: *A*, cm^3^ molecule^-1^ s^-1^; *n*, unitless; *E*, kJ/mol; $T_{0}$, K.

**Table S2** Fitting parameters for the high-pressure-limit rate constants (*k*_–1_) of the reverse reactions of R5-R8

| $k_{-1}$ | $E$ (kJ/mol) | $\ln A$ | $T_{0}$ (K) | $n$ |
| --- | --- | --- | --- | --- |
| *o*-add (R5) | 81.5 | 32.26 | -6.31 | -0.6357 |
| *m*-add (R6) | 81.6 | 31.99 | -4.292 | -0.5563 |
| *p*-add (R7) | 81.0 | 30.35 | -3.363 | -0.3679 |
| *ipso*-add (R8) | 83.7 | 32.27 | -3.14 | -0.73 |

Fitted by using $k=A\left( \frac{T}{300} \right)^{n}\exp\left[ -\frac{E\left( T+T_{0} \right)}{R\left( T^{2}+T_{0}^{2} \right)} \right]$; the unit of parameter *A* is $s^{-1}$; *R* is the gas constant.

**Table S3** The Arrhenius pre-exponential factor $A_{\infty}\left( T \right)$ (s^-1^) and Arrhenius activation energy $E_{a}\left( T \right)$ (kcal/mol) of the high-pressure-limit rate constants of reverse reactions of R5-R8. These are obtained using the fitting parameters of *k*_–1_.

| *T*/K | Reverse reactions of | | | | | | | | |
| --- | --- | --- | --- | --- | --- | --- | --- | --- | --- |
|  | *o*-add (R5) | | *m*-add (R6) | | *p*-add (R7) | | *ipso*-add (R8) | | |
|  | $A_{\infty}\left( T \right)$ | $E_{a}\left( T \right)$ | $A_{\infty}\left( T \right)$ | $E_{a}\left( T \right)$ | $A_{\infty}\left( T \right)$ | $E_{a}\left( T \right)$ | $A_{\infty}\left( T \right)$ | $E_{a}\left( T \right)$ | |
| 213 | 2.11E+13 | 18.45 | 1.61E+13 | 18.00 | 5.79E+12 | 18.56 | 3.27E+13 | | 18.86 |
| 225 | 2.23E+13 | 18.48 | 1.80E+13 | 18.05 | 6.00E+12 | 18.58 | 3.20E+13 | | 18.87 |
| 250 | 2.46E+13 | 18.53 | 2.17E+13 | 18.14 | 6.50E+12 | 18.63 | 3.32E+13 | | 18.89 |
| 298.15 | 2.73E+13 | 18.59 | 2.64E+13 | 18.24 | 7.13E+12 | 18.68 | 3.42E+13 | | 18.90 |
| 300 | 2.76E+13 | 18.59 | 2.65E+13 | 18.25 | 7.16E+12 | 18.68 | 3.41E+13 | | 18.90 |
| 325 | 2.83E+13 | 18.61 | 2.80E+13 | 18.28 | 7.34E+12 | 18.70 | 3.41E+13 | | 18.90 |
| 350 | 2.89E+13 | 18.62 | 2.90E+13 | 18.31 | 7.53E+12 | 18.71 | 3.40E+13 | | 18.89 |
| 375 | 2.93E+13 | 18.62 | 2.99E+13 | 18.33 | 7.63E+12 | 18.71 | 3.37E+13 | | 18.88 |
| 400 | 2.94E+13 | 18.62 | 3.03E+13 | 18.34 | 7.76E+12 | 18.72 | 3.33E+13 | | 18.87 |
| 425 | 2.94E+13 | 18.62 | 3.07E+13 | 18.34 | 7.73E+12 | 18.72 | 3.27E+13 | | 18.85 |
| 450 | 2.93E+13 | 18.62 | 3.07E+13 | 18.34 | 7.78E+12 | 18.72 | 3.20E+13 | | 18.83 |
| 475 | 2.90E+13 | 18.61 | 3.09E+13 | 18.34 | 7.71E+12 | 18.72 | 3.14E+13 | | 18.81 |
| 500 | 2.87E+13 | 18.60 | 3.08E+13 | 18.34 | 7.72E+12 | 18.71 | 3.06E+13 | | 18.78 |
| 550 | 2.82E+13 | 18.58 | 3.04E+13 | 18.32 | 7.63E+12 | 18.70 | 2.92E+13 | | 18.73 |
| 600 | 2.74E+13 | 18.55 | 2.96E+13 | 18.30 | 7.46E+12 | 18.68 | 2.80E+13 | | 18.68 |
| 650 | 2.69E+13 | 18.51 | 2.89E+13 | 18.27 | 7.40E+12 | 18.66 | 2.65E+13 | | 18.62 |
| 700 | 2.59E+13 | 18.48 | 2.81E+13 | 18.23 | 7.25E+12 | 18.64 | 2.54E+13 | | 18.57 |
| 800 | 2.46E+13 | 18.40 | 2.66E+13 | 18.15 | 6.97E+12 | 18.59 | 2.32E+13 | | 18.44 |
| 1000 | 2.20E+13 | 18.22 | 2.38E+13 | 17.96 | 6.45E+12 | 18.48 | 1.97E+13 | | 18.18 |
| 1200 | 1.98E+13 | 18.03 | 2.14E+13 | 17.75 | 6.08E+12 | 18.35 | 1.73E+13 | | 17.92 |
| 1400 | 1.84E+13 | 17.82 | 1.96E+13 | 17.53 | 5.78E+12 | 18.22 | 1.55E+13 | | 17.64 |
| 1600 | 1.72E+13 | 17.62 | 1.81E+13 | 17.30 | 5.51E+12 | 18.09 | 1.42E+13 | | 17.36 |
| 1800 | 1.62E+13 | 17.41 | 1.69E+13 | 17.06 | 5.31E+12 | 17.95 | 1.31E+13 | | 17.08 |
| 2000 | 1.54E+13 | 17.20 | 1.59E+13 | 16.82 | 5.17E+12 | 17.81 | 1.23E+13 | | 16.80 |
| 2200 | 1.48E+13 | 16.98 | 1.52E+13 | 16.58 | 5.05E+12 | 17.67 | 1.16E+13 | | 16.51 |
| 2300 | 1.44E+13 | 16.88 | 1.48E+13 | 16.46 | 5.00E+12 | 17.60 | 1.13E+13 | | 16.37 |
| 2400 | 1.43E+13 | 16.77 | 1.45E+13 | 16.34 | 4.90E+12 | 17.53 | 1.10E+13 | | 16.23 |
| 2500 | 1.40E+13 | 16.66 | 1.42E+13 | 16.22 | 4.85E+12 | 17.46 | 1.08E+13 | | 16.08 |

$E_{a}(T)$ is obtained by $E_{a}\left( T \right)=\frac{E\left( T^{4}+2T_{0}T^{3}-T_{0}^{2}T^{2} \right)}{\left( T^{2}+T_{0}^{2} \right)^{2}}+nRT$ where *E*, *T*_0_, and *n* are fitting parameters given in Table S2; $A_{\infty}(T)$ is obtained by $A_{\infty}\left( T \right)=k\left( T \right)\times\exp\left[ \frac{E_{a}\left( T \right)}{RT} \right]$.

**Table S4** Energy dependence factor of the density of states ${(F}_{E})$ as a function of temperature as calculated by the Whitten-Rabinovitch method

| *T*/K | *o*-add | *m*-add | *p*-add | *ipso*-add |
| --- | --- | --- | --- | --- |
|  | $F_{E}$ | $F_{E}$ | $F_{E}$ | $F_{E}$ |
| 213 | 1.287E+00 | 1.287E+00 | 1.286E+00 | 1.285E+00 |
| 225 | 1.308E+00 | 1.308E+00 | 1.307E+00 | 1.305E+00 |
| 250 | 1.353E+00 | 1.353E+00 | 1.352E+00 | 1.350E+00 |
| 298.15 | 1.449E+00 | 1.450E+00 | 1.449E+00 | 1.447E+00 |
| 300 | 1.453E+00 | 1.454E+00 | 1.453E+00 | 1.451E+00 |
| 325 | 1.509E+00 | 1.510E+00 | 1.508E+00 | 1.506E+00 |
| 350 | 1.569E+00 | 1.570E+00 | 1.568E+00 | 1.566E+00 |
| 375 | 1.634E+00 | 1.635E+00 | 1.633E+00 | 1.631E+00 |
| 400 | 1.704E+00 | 1.705E+00 | 1.703E+00 | 1.701E+00 |
| 425 | 1.780E+00 | 1.782E+00 | 1.779E+00 | 1.777E+00 |
| 450 | 1.862E+00 | 1.865E+00 | 1.862E+00 | 1.860E+00 |
| 475 | 1.953E+00 | 1.955E+00 | 1.952E+00 | 1.950E+00 |
| 500 | 2.051E+00 | 2.055E+00 | 2.051E+00 | 2.049E+00 |
| 550 | 2.279E+00 | 2.284E+00 | 2.278E+00 | 2.278E+00 |
| 600 | 2.558E+00 | 2.565E+00 | 2.557E+00 | 2.558E+00 |
| 650 | 2.906E+00 | 2.915E+00 | 2.902E+00 | 2.908E+00 |
| 700 | 3.345E+00 | 3.359E+00 | 3.340E+00 | 3.351E+00 |
| 800 | 4.664E+00 | 4.690E+00 | 4.645E+00 | 4.684E+00 |
| 1000 | 1.199E+01 | 1.211E+01 | 1.181E+01 | 1.217E+01 |
| 1200 | 5.087E+01 | 5.159E+01 | 4.884E+01 | 5.271E+01 |
| 1400 | 3.528E+02 | 3.581E+02 | 3.254E+02 | 3.761E+02 |
| 1600 | 3.358E+03 | 3.395E+03 | 2.945E+03 | 3.693E+03 |
| 1800 | 3.708E+04 | 3.721E+04 | 3.074E+04 | 4.210E+04 |
| 2000 | 4.286E+05 | 4.256E+05 | 3.343E+05 | 5.027E+05 |
| 2200 | 4.904E+06 | 4.806E+06 | 3.584E+06 | 5.942E+06 |
| 2300 | 1.636E+07 | 1.591E+07 | 1.156E+07 | 2.014E+07 |
| 2400 | 5.388E+07 | 5.201E+07 | 3.677E+07 | 6.744E+07 |
| 2500 | 1.749E+08 | 1.675E+08 | 1.153E+08 | 2.226E+08 |

**Table S5** Collision efficiency $\beta_{c}$ and collisional deactivation rate constant $k_{c}$ (cm^3^ molecule^-1^ s^-1^) as functions of temperature

| *T*/K | *o*-add | | *m*-add | | *p*-add | | *ipso*-add | |
| --- | --- | --- | --- | --- | --- | --- | --- | --- |
|  | $\beta_{c}$ | $k_{c}$ | $\beta_{c}$ | $k_{c}$ | $\beta_{c}$ | $k_{c}$ | $\beta_{c}$ | $k_{c}$ |
| 213 | 3.06E-01 | 1.67E-10 | 3.05E-01 | 1.67E-10 | 3.05E-01 | 1.67E-10 | 3.06E-01 | 1.67E-10 |
| 225 | 2.92E-01 | 1.61E-10 | 2.92E-01 | 1.61E-10 | 2.92E-01 | 1.61E-10 | 2.92E-01 | 1.61E-10 |
| 250 | 2.67E-01 | 1.49E-10 | 2.67E-01 | 1.49E-10 | 2.67E-01 | 1.49E-10 | 2.67E-01 | 1.49E-10 |
| 298.15 | 2.27E-01 | 1.29E-10 | 2.27E-01 | 1.29E-10 | 2.27E-01 | 1.30E-10 | 2.27E-01 | 1.30E-10 |
| 300 | 2.26E-01 | 1.29E-10 | 2.25E-01 | 1.29E-10 | 2.25E-01 | 1.29E-10 | 2.26E-01 | 1.29E-10 |
| 325 | 2.08E-01 | 1.20E-10 | 2.08E-01 | 1.20E-10 | 2.08E-01 | 1.20E-10 | 2.08E-01 | 1.20E-10 |
| 350 | 1.92E-01 | 1.12E-10 | 1.91E-01 | 1.12E-10 | 1.92E-01 | 1.12E-10 | 1.92E-01 | 1.12E-10 |
| 375 | 1.77E-01 | 1.05E-10 | 1.77E-01 | 1.05E-10 | 1.77E-01 | 1.05E-10 | 1.77E-01 | 1.05E-10 |
| 400 | 1.64E-01 | 9.81E-11 | 1.63E-01 | 9.80E-11 | 1.64E-01 | 9.82E-11 | 1.64E-01 | 9.81E-11 |
| 425 | 1.51E-01 | 9.17E-11 | 1.51E-01 | 9.17E-11 | 1.51E-01 | 9.18E-11 | 1.51E-01 | 9.18E-11 |
| 450 | 1.40E-01 | 8.58E-11 | 1.40E-01 | 8.57E-11 | 1.40E-01 | 8.59E-11 | 1.40E-01 | 8.58E-11 |
| 475 | 1.29E-01 | 8.01E-11 | 1.29E-01 | 8.00E-11 | 1.29E-01 | 8.02E-11 | 1.29E-01 | 8.02E-11 |
| 500 | 1.19E-01 | 7.48E-11 | 1.19E-01 | 7.47E-11 | 1.19E-01 | 7.49E-11 | 1.19E-01 | 7.48E-11 |
| 550 | 1.02E-01 | 6.50E-11 | 1.01E-01 | 6.49E-11 | 1.02E-01 | 6.50E-11 | 1.02E-01 | 6.50E-11 |
| 600 | 8.62E-02 | 5.61E-11 | 8.59E-02 | 5.60E-11 | 8.61E-02 | 5.61E-11 | 8.61E-02 | 5.61E-11 |
| 650 | 7.24E-02 | 4.80E-11 | 7.22E-02 | 4.79E-11 | 7.25E-02 | 4.80E-11 | 7.23E-02 | 4.81E-11 |
| 700 | 6.03E-02 | 3.91E-11 | 6.01E-02 | 3.90E-11 | 6.04E-02 | 3.90E-11 | 6.02E-02 | 3.92E-11 |
| 800 | 4.01E-02 | 2.70E-11 | 3.99E-02 | 2.68E-11 | 4.02E-02 | 2.69E-11 | 3.99E-02 | 2.71E-11 |
| 1000 | 1.38E-02 | 9.85E-12 | 1.36E-02 | 9.76E-12 | 1.40E-02 | 9.71E-12 | 1.36E-02 | 9.99E-12 |
| 1200 | 2.94E-03 | 2.19E-12 | 2.86E-03 | 2.16E-12 | 3.02E-03 | 2.11E-12 | 2.80E-03 | 2.27E-12 |
| 1400 | 3.83E-04 | 2.93E-13 | 3.66E-04 | 2.89E-13 | 4.02E-04 | 2.75E-13 | 3.49E-04 | 3.17E-13 |
| 1600 | 3.65E-05 | 2.84E-14 | 3.42E-05 | 2.81E-14 | 3.94E-05 | 2.58E-14 | 3.15E-05 | 3.24E-14 |
| 1800 | 3.03E-06 | 2.38E-15 | 2.79E-06 | 2.37E-15 | 3.37E-06 | 2.10E-15 | 2.46E-06 | 2.87E-15 |
| 2000 | 2.44E-07 | 1.92E-16 | 2.20E-07 | 1.93E-16 | 2.80E-07 | 1.63E-16 | 1.86E-07 | 2.46E-16 |
| 2200 | 2.01E-08 | 1.57E-17 | 1.77E-08 | 1.60E-17 | 2.37E-08 | 1.30E-17 | 1.43E-08 | 2.15E-17 |
| 2300 | 5.86E-09 | 4.57E-18 | 5.11E-09 | 4.69E-18 | 7.04E-09 | 3.71E-18 | 4.04E-09 | 6.46E-18 |
| 2400 | 1.74E-09 | 1.35E-18 | 1.50E-09 | 1.40E-18 | 2.12E-09 | 1.08E-18 | 1.16E-09 | 1.97E-18 |
| 2500 | 5.24E-10 | 4.03E-19 | 4.47E-10 | 4.21E-19 | 6.49E-10 | 3.17E-19 | 3.36E-10 | 6.12E-19 |

**Geometries and absolute energies in hartrees of species involved in the abstraction reactions as optimized by the M06-2X/MG3S method**

C_6_H_5_CH_3_

--------------------------------------------------------------------------------------------------------

Center Atomic Atomic Coordinates (Angstroms)

Number Number Type X Y Z

--------------------------------------------------------------------------------------------------------

1 6 0 -0.907464 0.003270 -0.010360

2 6 0 -0.195811 -1.194239 -0.008076

3 6 0 1.191138 -1.200173 0.001837

4 6 0 1.893351 -0.002342 0.007716

5 6 0 1.196533 1.197128 0.001795

6 6 0 -0.191859 1.196779 -0.007980

7 1 0 -0.737444 -2.132463 -0.016666

8 1 0 1.724858 -2.141124 0.001570

9 1 0 2.974675 -0.004678 0.012673

10 1 0 1.733635 2.136142 0.001445

11 1 0 -0.729244 2.137181 -0.016494

12 6 0 -2.412076 0.001157 0.008402

13 1 0 -2.811692 -0.807462 -0.602311

14 1 0 -2.785345 -0.139435 1.024255

15 1 0 -2.812318 0.942365 -0.364469

--------------------------------------------------------------------------------------------------------

*E* = –271.5235858 a.u.

OH

--------------------------------------------------------------------------------------------------------

Center Atomic Atomic Coordinates (Angstroms)

Number Number Type X Y Z

--------------------------------------------------------------------------------------------------------

1 8 0 0.000000 0.000000 0.107877

2 1 0 0.000000 0.000000 -0.863018

--------------------------------------------------------------------------------------------------------

*E* = –75.729135 a.u.

TS-abs-CH3

--------------------------------------------------------------------------------------------------------

Center Atomic Atomic Coordinates (Angstroms)

Number Number Type X Y Z

--------------------------------------------------------------------------------------------------------

1 6 0 0.318980 -0.000077 -0.566027

2 6 0 -0.345439 -1.198982 -0.306446

3 6 0 -1.637917 -1.200422 0.195881

4 6 0 -2.288261 0.000078 0.449043

5 6 0 -1.637845 1.200501 0.195699

6 6 0 -0.345369 1.198906 -0.306628

7 1 0 0.158955 -2.136880 -0.503737

8 1 0 -2.139404 -2.139298 0.387714

9 1 0 -3.296780 0.000136 0.839019

10 1 0 -2.139277 2.139436 0.387387

11 1 0 0.159081 2.136744 -0.504065

12 6 0 1.721754 -0.000152 -1.062072

13 1 0 1.978916 0.895859 -1.622666

14 1 0 2.446397 -0.000075 -0.160186

15 1 0 1.978886 -0.896278 -1.622494

16 8 0 3.000361 0.000132 1.195928

17 1 0 2.134917 0.000188 1.634902

--------------------------------------------------------------------------------------------------------

*E* = –347.2506097 a.u.

TS-abs-*m*

--------------------------------------------------------------------------------------------------------

Center Atomic Atomic Coordinates (Angstroms)

Number Number Type X Y Z

--------------------------------------------------------------------------------------------------------

1 6 0 -0.437366 1.846337 0.026524

2 6 0 0.865651 1.369499 -0.059380

3 6 0 1.045961 0.002125 -0.113844

4 6 0 0.000903 -0.896147 -0.071505

5 6 0 -1.307760 -0.416976 0.010224

6 6 0 -1.505735 0.960220 0.061897

7 1 0 -0.618621 2.912016 0.069519

8 1 0 1.709219 2.044711 -0.086974

9 1 0 2.161690 -0.427470 -0.213386

10 1 0 3.358554 -0.873067 0.964152

11 8 0 3.360022 -0.892423 -0.004128

12 1 0 0.190328 -1.961698 -0.107763

13 1 0 -2.515500 1.344738 0.134175

14 6 0 -2.470464 -1.371619 0.018101

15 1 0 -2.731170 -1.667731 -0.999244

16 1 0 -2.231280 -2.277626 0.572975

17 1 0 -3.350541 -0.915120 0.467464

--------------------------------------------------------------------------------------------------------

*E* = –347.2440509 a.u.

TS-abs-*o*

--------------------------------------------------------------------------------------------------------

Center Atomic Atomic Coordinates (Angstroms)

Number Number Type X Y Z

--------------------------------------------------------------------------------------------------------

1 6 0 1.815589 -1.191642 0.005734

2 6 0 0.465872 -1.510049 -0.073365

3 6 0 -0.452850 -0.478665 -0.096562

4 6 0 -0.111584 0.860178 -0.030422

5 6 0 1.252566 1.151817 0.046253

6 6 0 2.201952 0.141995 0.065067

7 1 0 2.557962 -1.977804 0.018621

8 1 0 0.138026 -2.539234 -0.125847

9 1 0 -1.614202 -0.757361 -0.201014

10 1 0 1.564942 2.188497 0.091112

11 1 0 3.251607 0.395774 0.126243

12 6 0 -1.145306 1.952301 -0.039395

13 1 0 -0.917600 2.691641 -0.806943

14 1 0 -2.136583 1.546775 -0.228767

15 1 0 -1.163539 2.470885 0.919994

16 1 0 -2.848234 -1.153559 0.960756

17 8 0 -2.873726 -1.052654 -0.002252

--------------------------------------------------------------------------------------------------------

*E* = –347.245064 a.u.

TS-abs-*p*

--------------------------------------------------------------------------------------------------------

Center Atomic Atomic Coordinates (Angstroms)

Number Number Type X Y Z

--------------------------------------------------------------------------------------------------------

1 6 0 -0.839436 1.203865 0.062198

2 6 0 0.549735 1.222622 0.080326

3 6 0 1.222768 0.017789 0.046485

4 6 0 0.566175 -1.192781 -0.023296

5 6 0 -0.824914 -1.190718 -0.040516

6 6 0 -1.541931 0.001544 0.004345

7 1 0 -1.386408 2.138568 0.091586

8 1 0 1.090323 2.158056 0.125115

9 1 0 2.424670 0.024360 0.092071

10 1 0 -1.358957 -2.131592 -0.092107

11 1 0 3.640536 0.066750 -1.141343

12 8 0 3.692102 0.043029 -0.174651

13 6 0 -3.046810 -0.002341 0.011772

14 1 0 -3.440645 -0.938880 -0.378532

15 1 0 -3.426969 0.121570 1.027038

16 1 0 -3.444626 0.814195 -0.589325

17 1 0 1.118352 -2.121832 -0.058887

--------------------------------------------------------------------------------------------------------

*E* = –347.2437337 a.u.

*m*-C_6_H_4_CH_3_

--------------------------------------------------------------------------------------------------------

Center Atomic Atomic Coordinates (Angstroms)

Number Number Type X Y Z

--------------------------------------------------------------------------------------------------------

1 6 0 -1.114499 -1.272675 0.002253

2 6 0 -1.941319 -0.180139 0.006885

3 6 0 -1.313609 1.064287 0.001461

4 6 0 0.073074 1.151697 -0.006876

5 6 0 0.875121 0.013376 -0.009090

6 6 0 0.252313 -1.241318 -0.006980

7 1 0 -3.018807 -0.267233 0.011320

8 1 0 -1.910224 1.967277 0.001010

9 1 0 0.544470 2.126146 -0.013939

10 6 0 2.375743 0.115543 0.006929

11 1 0 2.766534 -0.098688 1.002904

12 1 0 2.825179 -0.599426 -0.681364

13 1 0 2.705338 1.114226 -0.273200

14 1 0 0.846565 -2.146928 -0.014226

--------------------------------------------------------------------------------------------------------

*E* = –270.8378732 a.u.

*o*-C_6_H_4_CH_3_

--------------------------------------------------------------------------------------------------------

Center Atomic Atomic Coordinates (Angstroms)

Number Number Type X Y Z

--------------------------------------------------------------------------------------------------------

1 6 0 1.216636 -1.227703 -0.000002

2 6 0 1.883568 -0.000905 -0.000004

3 6 0 1.150877 1.177107 -0.000004

4 6 0 -0.239352 1.148395 -0.000002

5 6 0 -0.938694 -0.061044 0.000001

6 6 0 -0.150122 -1.188260 0.000000

7 1 0 1.763357 -2.161198 -0.000003

8 1 0 2.964961 0.026556 -0.000006

9 1 0 1.663746 2.129056 -0.000007

10 1 0 -0.797965 2.077003 -0.000004

11 6 0 -2.441296 -0.121828 0.000009

12 1 0 -2.807468 -0.652461 -0.878383

13 1 0 -2.807462 -0.652331 0.878482

14 1 0 -2.868878 0.878791 -0.000062

--------------------------------------------------------------------------------------------------------

*E* = –270.8379161 a.u.

*p*-C_6_H_4_CH_3_

--------------------------------------------------------------------------------------------------------

Center Atomic Atomic Coordinates (Angstroms)

Number Number Type X Y Z

--------------------------------------------------------------------------------------------------------

1 6 0 -1.271860 1.209928 0.001338

2 6 0 -1.897644 -0.006624 0.004511

3 6 0 -1.257824 -1.219134 0.001374

4 6 0 0.134594 -1.197167 -0.004074

5 6 0 0.837442 0.007651 -0.005382

6 6 0 0.124107 1.202616 -0.003969

7 1 0 -1.821597 2.141558 0.000989

8 1 0 -1.798701 -2.155920 0.001118

9 1 0 0.682865 -2.132195 -0.008713

10 1 0 0.661277 2.143450 -0.008536

11 6 0 2.343241 0.003789 0.004132

12 1 0 2.740649 1.003404 -0.160495

13 1 0 2.725070 -0.355752 0.960586

14 1 0 2.738100 -0.650888 -0.772533

--------------------------------------------------------------------------------------------------------

*E* = –270.8370967 a.u.

C_6_H_5_CH_2_

--------------------------------------------------------------------------------------------------------

Center Atomic Atomic Coordinates (Angstroms)

Number Number Type X Y Z

--------------------------------------------------------------------------------------------------------

1 6 0 0.000000 1.204544 -1.127521

2 6 0 0.000000 0.000000 -1.828200

3 6 0 0.000000 -1.204544 -1.127521

4 6 0 0.000000 -1.209907 0.251159

5 6 0 0.000000 0.000000 0.984907

6 6 0 0.000000 1.209907 0.251159

7 1 0 0.000000 2.141477 -1.667918

8 1 0 0.000000 0.000000 -2.909080

9 1 0 0.000000 -2.141477 -1.667918

10 1 0 0.000000 -2.147391 0.792422

11 1 0 0.000000 2.147391 0.792422

12 6 0 0.000000 0.000000 2.390897

13 1 0 0.000000 0.926088 2.945394

14 1 0 0.000000 -0.926088 2.945394

--------------------------------------------------------------------------------------------------------

*E* = –270.8707327 a.u.

H_2_O

--------------------------------------------------------------------------------------------------------

Center Atomic Atomic Coordinates (Angstroms)

Number Number Type X Y Z

--------------------------------------------------------------------------------------------------------

1 8 0 0.000000 0.000000 0.116211

2 1 0 0.000000 0.761632 -0.464846

3 1 0 0.000000 -0.761632 -0.464846

--------------------------------------------------------------------------------------------------------

*E* = –76.4248595 a.u.

Pre-Reactive Complex

--------------------------------------------------------------------------------------------------------

Center Atomic Atomic Coordinates (Angstroms)

Number Number Type X Y Z

--------------------------------------------------------------------------------------------------------

1 6 0 0.072103 -1.017992 -0.682123

2 6 0 -1.311018 -0.902565 -0.741849

3 6 0 -1.987661 -0.142666 0.202006

4 6 0 -1.271169 0.487850 1.213848

5 6 0 0.109287 0.362078 1.273175

6 6 0 0.802801 -0.383489 0.321310

7 1 0 0.595690 -1.598896 -1.430973

8 1 0 -1.857683 -1.399072 -1.531962

9 1 0 -3.063808 -0.047817 0.156373

10 1 0 -1.790523 1.075326 1.959083

11 1 0 0.661490 0.859637 2.060673

12 6 0 2.303490 -0.454630 0.340629

13 1 0 2.659399 -1.402703 -0.059238

14 1 0 2.718573 0.345228 -0.275145

15 1 0 2.691922 -0.337022 1.350838

16 8 0 0.481071 1.615423 -1.724742

17 1 0 -0.310793 1.602323 -1.159603

--------------------------------------------------------------------------------------------------------

*E* = –347.2622099 a.u.

**Geometries and absolute energies in hartrees of species involved in the addition reactions as optimized by M08-SO/MG3S (used in CVT/SCT)**

C_6_H_5_CH_3_

--------------------------------------------------------------------------------------------------------

Center Atomic Atomic Coordinates (Angstroms)

Number Number Type X Y Z

--------------------------------------------------------------------------------------------------------

1 6 0 0.910727 0.002301 -0.009422

2 6 0 0.193437 1.201110 -0.007847

3 6 0 -1.200573 1.202544 0.001716

4 6 0 -1.900909 -0.001493 0.007643

5 6 0 -1.196642 -1.204658 0.001752

6 6 0 0.196041 -1.199603 -0.007911

7 1 0 0.735815 2.146371 -0.016781

8 1 0 -1.740386 2.147532 0.001028

9 1 0 -2.988789 -0.003188 0.012454

10 1 0 -1.734238 -2.150924 0.001141

11 1 0 0.740906 -2.143601 -0.016906

12 6 0 2.421462 0.000937 0.008231

13 1 0 2.822406 0.946973 -0.373174

14 1 0 2.799448 -0.134474 1.030679

15 1 0 2.823578 -0.815514 -0.603418

--------------------------------------------------------------------------------------------------------

*E* = –271.4319399 a.u.

OH

--------------------------------------------------------------------------------------------------------

Center Atomic Atomic Coordinates (Angstroms)

Number Number Type X Y Z

--------------------------------------------------------------------------------------------------------

1 8 0 0.000000 0.000000 0.108349

2 1 0 0.000000 0.000000 -0.866791

--------------------------------------------------------------------------------------------------------

*E* = –75.7091708 a.u.

TS-add-*ipso*

--------------------------------------------------------------------------------------------------------

Center Atomic Atomic Coordinates (Angstroms)

Number Number Type X Y Z

--------------------------------------------------------------------------------------------------------

1 6 0 0.020437 -1.217293 -0.273376

2 6 0 1.392808 -1.213331 -0.098639

3 6 0 2.086110 0.000000 -0.005446

4 6 0 1.392807 1.213331 -0.098641

5 6 0 0.020436 1.217292 -0.273378

6 6 0 -0.721315 -0.000001 -0.315274

7 1 0 -0.526826 -2.155900 -0.344232

8 1 0 1.934846 -2.154473 -0.036873

9 1 0 3.165401 0.000001 0.131604

10 1 0 1.934845 2.154474 -0.036877

11 1 0 -0.526827 2.155899 -0.344236

12 6 0 -2.142493 -0.000001 -0.821155

13 1 0 -2.671171 -0.887679 -0.458799

14 1 0 -2.671171 0.887677 -0.458801

15 1 0 -2.155872 -0.000002 -1.918994

16 8 0 -1.293645 0.000003 1.593722

17 1 0 -0.426794 -0.000002 2.032881

--------------------------------------------------------------------------------------------------------

*E* = –347.1448722 a.u.

TS-add-*m*

--------------------------------------------------------------------------------------------------------

Center Atomic Atomic Coordinates (Angstroms)

Number Number Type X Y Z

--------------------------------------------------------------------------------------------------------

1 6 0 0.868227 1.209578 0.296215

2 6 0 -0.422111 1.716649 0.120594

3 6 0 -1.420152 0.912886 -0.407594

4 6 0 -1.142783 -0.444202 -0.716848

5 6 0 0.192983 -0.914666 -0.591770

6 6 0 1.191075 -0.112147 -0.064788

7 1 0 1.646804 1.850105 0.710703

8 1 0 -0.639350 2.747071 0.393609

9 1 0 -2.428539 1.293504 -0.548303

10 1 0 -1.849995 -1.010400 -1.315236

11 1 0 0.415633 -1.941251 -0.878009

12 6 0 2.598210 -0.627955 0.122522

13 1 0 3.325953 0.024300 -0.376442

14 1 0 2.863627 -0.663935 1.187142

15 1 0 2.705023 -1.638062 -0.287339

16 8 0 -1.973699 -1.283556 0.876210

17 1 0 -1.442256 -0.833733 1.554209

--------------------------------------------------------------------------------------------------------

*E* = –347.1427513 a.u.

TS-add-*o*

--------------------------------------------------------------------------------------------------------

Center Atomic Atomic Coordinates (Angstroms)

Number Number Type X Y Z

--------------------------------------------------------------------------------------------------------

1 6 0 0.135404 -1.442938 0.297481

2 6 0 1.504503 -1.165037 0.359564

3 6 0 2.005864 0.055236 -0.112076

4 6 0 1.137462 0.995877 -0.638254

5 6 0 -0.259515 0.751222 -0.647260

6 6 0 -0.758154 -0.515799 -0.222801

7 1 0 -0.239861 -2.399054 0.659749

8 1 0 2.186214 -1.907033 0.770651

9 1 0 3.073956 0.256500 -0.073038

10 1 0 1.508460 1.946091 -1.015077

11 1 0 -0.908310 1.398450 -1.232349

12 6 0 -2.237265 -0.766678 -0.281574

13 1 0 -2.491665 -1.764366 0.091676

14 1 0 -2.751730 -0.012145 0.329010

15 1 0 -2.611232 -0.672975 -1.309849

16 8 0 -0.844144 1.780670 0.958340

17 1 0 -0.182473 1.437874 1.582018

--------------------------------------------------------------------------------------------------------

*E* = –347.1463225 a.u.

TS-add-*p*

--------------------------------------------------------------------------------------------------------

Center Atomic Atomic Coordinates (Angstroms)

Number Number Type X Y Z

--------------------------------------------------------------------------------------------------------

1 6 0 -0.607689 -1.208398 -0.177244

2 6 0 0.732686 -1.219406 -0.516092

3 6 0 1.454158 -0.001829 -0.634401

4 6 0 0.735626 1.216941 -0.517986

5 6 0 -0.605528 1.209357 -0.178415

6 6 0 -1.298838 0.001794 0.006541

7 1 0 -1.144704 -2.148802 -0.057454

8 1 0 1.258930 -2.160462 -0.656652

9 1 0 2.436543 -0.003319 -1.095789

10 1 0 1.263713 2.156725 -0.660080

11 1 0 -1.140290 2.151058 -0.059542

12 6 0 -2.752055 0.001157 0.405058

13 1 0 -3.281840 -0.853433 -0.031934

14 1 0 -2.854014 -0.071343 1.497104

15 1 0 -3.251525 0.922997 0.086043

16 8 0 2.394642 0.000671 1.120688

17 1 0 1.605890 0.003507 1.688036

--------------------------------------------------------------------------------------------------------

*E* = –347.1437903 a.u.

*ipso*-C_6_H_5_OHCH_3_

--------------------------------------------------------------------------------------------------------

Center Atomic Atomic Coordinates (Angstroms)

Number Number Type X Y Z

--------------------------------------------------------------------------------------------------------

1 6 0 0.037040 1.248828 -0.035413

2 6 0 1.395905 1.227711 0.025364

3 6 0 2.108782 0.000001 0.074179

4 6 0 1.395904 -1.227707 0.025442

5 6 0 0.037036 -1.248829 -0.035333

6 6 0 -0.806078 -0.000001 -0.038095

7 1 0 -0.511595 2.189683 -0.085708

8 1 0 1.950898 2.164507 0.028411

9 1 0 3.194773 0.000003 0.123247

10 1 0 1.950892 -2.164506 0.028552

11 1 0 -0.511594 -2.189691 -0.085553

12 6 0 -1.760330 0.000043 1.166277

13 1 0 -2.397752 0.891960 1.125760

14 1 0 -2.397725 -0.891897 1.125852

15 1 0 -1.193294 0.000102 2.103640

16 8 0 -1.675677 -0.000030 -1.184835

17 1 0 -1.128747 -0.000206 -1.980038

--------------------------------------------------------------------------------------------------------

*E* = –347.1766718 a.u.

*m*-C_6_H_5_OHCH_3_

--------------------------------------------------------------------------------------------------------

Center Atomic Atomic Coordinates (Angstroms)

Number Number Type X Y Z

--------------------------------------------------------------------------------------------------------

1 6 0 1.024036 1.216138 0.140118

2 6 0 -0.271394 1.785499 0.067958

3 6 0 -1.365004 1.017117 -0.192829

4 6 0 -1.268418 -0.464899 -0.414284

5 6 0 0.137684 -0.981624 -0.299730

6 6 0 1.211949 -0.189770 -0.036426

7 1 0 1.885902 1.847313 0.351602

8 1 0 -0.388880 2.855498 0.232567

9 1 0 -2.362831 1.451187 -0.234125

10 1 0 -1.662163 -0.705924 -1.417870

11 1 0 0.264334 -2.057598 -0.425420

12 6 0 2.605798 -0.758685 0.082122

13 1 0 3.272082 -0.313656 -0.668516

14 1 0 3.033293 -0.541036 1.069633

15 1 0 2.602416 -1.844554 -0.061087

16 8 0 -2.153948 -1.176365 0.462773

17 1 0 -1.860475 -1.022961 1.369452

--------------------------------------------------------------------------------------------------------

*E* = –347.1748088 a.u.

*o*-C_6_H_5_OHCH_3_

--------------------------------------------------------------------------------------------------------

Center Atomic Atomic Coordinates (Angstroms)

Number Number Type X Y Z

--------------------------------------------------------------------------------------------------------

1 6 0 2.028560 0.216515 0.051478

2 6 0 1.094285 1.163071 0.334311

3 6 0 -0.375333 0.860257 0.371634

4 6 0 -0.701637 -0.591872 0.111402

5 6 0 0.283634 -1.496338 -0.165191

6 6 0 1.651328 -1.130030 -0.200444

7 1 0 3.081943 0.489528 0.014245

8 1 0 1.379296 2.197590 0.520704

9 1 0 0.007173 -2.532103 -0.364619

10 1 0 2.409082 -1.875727 -0.427485

11 6 0 -2.154263 -0.957405 0.169887

12 1 0 -2.556886 -0.772187 1.177745

13 1 0 -2.737786 -0.332036 -0.518844

14 1 0 -2.312970 -2.012339 -0.079259

15 8 0 -1.091644 1.713876 -0.537772

16 1 0 -0.706539 1.598982 -1.415738

17 1 0 -0.789599 1.142094 1.356960

--------------------------------------------------------------------------------------------------------

*E* = –347.1774424 a.u.

*p*-C_6_H_5_OHCH_3_

--------------------------------------------------------------------------------------------------------

Center Atomic Atomic Coordinates (Angstroms)

Number Number Type X Y Z

--------------------------------------------------------------------------------------------------------

1 6 0 0.639170 1.226174 -0.072519

2 6 0 -0.707870 1.248462 -0.268469

3 6 0 -1.532695 -0.001756 -0.382758

4 6 0 -0.699736 -1.247621 -0.270050

5 6 0 0.642515 -1.217452 -0.074636

6 6 0 1.368924 0.009678 0.020797

7 1 0 1.184536 2.165926 0.021388

8 1 0 -1.247855 2.192434 -0.326789

9 1 0 -2.068447 -0.003536 -1.347846

10 1 0 -1.234936 -2.194359 -0.329216

11 1 0 1.194244 -2.153970 0.018385

12 6 0 2.856117 -0.002543 0.221009

13 1 0 3.251074 1.012202 0.342561

14 1 0 3.133536 -0.587385 1.109765

15 1 0 3.367124 -0.465169 -0.636452

16 8 0 -2.597138 -0.006974 0.580868

17 1 0 -2.200710 0.000001 1.461019

--------------------------------------------------------------------------------------------------------

*E* = –347.1757001 a.u.

Pre-Reactive Complex

--------------------------------------------------------------------------------------------------------

Center Atomic Atomic Coordinates (Angstroms)

Number Number Type X Y Z

--------------------------------------------------------------------------------------------------------

1 6 0 -0.017305 -1.316491 -0.437416

2 6 0 -1.391635 -1.115704 -0.542860

3 6 0 -1.998489 -0.042360 0.114215

4 6 0 -1.219058 0.822553 0.878220

5 6 0 0.160079 0.624233 0.972143

6 6 0 0.780869 -0.446617 0.313251

7 1 0 0.450055 -2.154111 -0.953933

8 1 0 -1.994275 -1.800182 -1.136786

9 1 0 -3.072315 0.111965 0.034722

10 1 0 -1.679623 1.661936 1.394879

11 1 0 0.767737 1.311360 1.559241

12 6 0 2.277589 -0.618246 0.372552

13 1 0 2.569897 -1.645092 0.125819

14 1 0 2.753802 0.058236 -0.349841

15 1 0 2.666246 -0.375028 1.368420

16 8 0 0.758619 1.759942 -1.317728

17 1 0 -0.082772 1.307177 -1.521329

--------------------------------------------------------------------------------------------------------

*E* = –347.1507544 a.u.

**Conformers with Higher Energies (used in MS-T)**

TS-abs-*p* Conformer1

---------------------------------------------------------------------------------------------------------

Center Atomic Atomic Coordinates (Angstroms)

Number Number Type X Y Z

-------------------------------------------------------------------------------------------------------

1 6 0 -0.836539 1.198422 -0.074260

2 6 0 0.546694 1.208107 -0.214242

3 6 0 1.207419 -0.001119 -0.282958

4 6 0 0.546453 -1.209579 -0.203609

5 6 0 -0.836766 -1.198392 -0.063701

6 6 0 -1.542580 0.000411 0.005411

7 1 0 -1.374740 2.137399 -0.028968

8 1 0 1.089765 2.140889 -0.279667

9 1 0 2.399856 -0.001898 -0.440277

10 1 0 -1.375157 -2.136829 -0.010128

11 1 0 3.726671 0.001207 0.673182

12 8 0 3.687009 -0.001583 -0.294369

13 6 0 -3.036182 0.001331 0.189779

14 1 0 -3.488342 -0.882761 -0.256571

15 1 0 -3.293297 0.004619 1.250328

16 1 0 -3.487924 0.882886 -0.261965

17 1 0 1.089336 -2.143007 -0.260876

-------------------------------------------------------------------------------------------------------

*E* = –347.2437406 a.u.

TS-abs-CH3 Conformer1

------------------------------------------------------------------------------------------------------

Center Atomic Atomic Coordinates (Angstroms)

Number Number Type X Y Z

------------------------------------------------------------------------------------------------------

1 6 0 0.185771 -0.204880 -0.591782

2 6 0 -0.725933 -1.260176 -0.606743

3 6 0 -2.007385 -1.097845 -0.104129

4 6 0 -2.397600 0.124858 0.426089

5 6 0 -1.497988 1.182458 0.449872

6 6 0 -0.217165 1.016945 -0.053410

7 1 0 -0.423534 -2.215170 -1.018088

8 1 0 -2.703184 -1.925565 -0.126396

9 1 0 -3.397364 0.253193 0.817616

10 1 0 -1.795609 2.137689 0.860622

11 1 0 0.482924 1.842821 -0.032257

12 6 0 1.568206 -0.387830 -1.110041

13 1 0 2.017730 0.538985 -1.459716

14 1 0 2.229158 -0.746684 -0.239851

15 1 0 1.636046 -1.157832 -1.875537

16 8 0 3.267257 -1.238476 0.716331

17 1 0 3.987095 -1.362203 0.078812

------------------------------------------------------------------------------------------------------

*E* = –347.2492266 a.u.

*ipso*-C_6_H_5_OHCH_3_ Conformer1

------------------------------------------------------------------------------------------------------

Center Atomic Atomic Coordinates (Angstroms)

Number Number Type X Y Z

------------------------------------------------------------------------------------------------------

1 6 0 0.011932 1.242980 -0.082120

2 6 0 1.365524 1.223144 0.031548

3 6 0 2.079433 -0.002353 0.115429

4 6 0 1.372615 -1.231565 0.034741

5 6 0 0.017759 -1.253445 -0.069279

6 6 0 -0.827904 -0.004857 -0.055954

7 1 0 -0.536727 2.179264 -0.179756

8 1 0 1.918172 2.161025 0.040044

9 1 0 3.162861 0.000673 0.203325

10 1 0 1.929567 -2.167028 0.044787

11 1 0 -0.520468 -2.200571 -0.140207

12 6 0 -1.714105 0.001495 1.208406

13 1 0 -2.352485 0.893883 1.204707

14 1 0 -2.352733 -0.894443 1.223120

15 1 0 -1.096167 0.000152 2.113603

16 8 0 -1.676487 0.051567 -1.212626

17 1 0 -2.338800 -0.647573 -1.152791

------------------------------------------------------------------------------------------------------

*E* = –347.1724142 a.u.

*m*-C_6_H_5_OHCH_3_ Conformer1

------------------------------------------------------------------------------------------------------

Center Atomic Atomic Coordinates (Angstroms)

Number Number Type X Y Z

------------------------------------------------------------------------------------------------------

1 6 0 1.041821 1.227012 0.117324

2 6 0 -0.241072 1.809903 0.067896

3 6 0 -1.345038 1.054134 -0.150901

4 6 0 -1.268926 -0.424631 -0.364424

5 6 0 0.120124 -0.958985 -0.247639

6 6 0 1.205372 -0.179105 -0.033087

7 1 0 1.910913 1.846021 0.294499

8 1 0 -0.340484 2.877420 0.215494

9 1 0 -2.328238 1.508349 -0.182729

10 1 0 -1.639202 -0.651133 -1.378577

11 1 0 0.222007 -2.033469 -0.338131

12 6 0 2.588669 -0.760770 0.069091

13 1 0 3.241794 -0.340322 -0.696785

14 1 0 3.032416 -0.529957 1.038271

15 1 0 2.571666 -1.841851 -0.051090

16 8 0 -2.084775 -1.148028 0.564600

17 1 0 -3.002623 -0.900725 0.427770

------------------------------------------------------------------------------------------------------

*E* = –347.17096372 a.u.

*o*-C_6_H_5_OHCH_3_ Conformer1

------------------------------------------------------------------------------------------------------

Center Atomic Atomic Coordinates (Angstroms)

Number Number Type X Y Z

------------------------------------------------------------------------------------------------------

1 6 0 -0.378672 0.821882 0.338448

2 6 0 1.075874 1.157243 0.213565

3 6 0 2.027750 0.207096 0.023093

4 6 0 1.677336 -1.162265 -0.119059

5 6 0 0.312328 -1.541223 -0.111885

6 6 0 -0.688148 -0.632247 0.070885

7 1 0 -0.697263 1.043946 1.382078

8 1 0 1.334145 2.211451 0.295361

9 1 0 3.075099 0.497589 -0.037666

10 1 0 2.450055 -1.911824 -0.269475

11 1 0 0.051399 -2.587460 -0.274248

12 6 0 -2.141098 -1.001364 0.045522

13 1 0 -2.652136 -0.651758 0.957375

14 1 0 -2.647463 -0.531421 -0.810405

15 1 0 -2.277745 -2.085516 -0.031117

16 8 0 -1.113644 1.696085 -0.529325

17 1 0 -2.024959 1.760980 -0.224101

------------------------------------------------------------------------------------------------------

*E* = –347.1736506 a.u.

*p*-C_6_H_5_OHCH_3_ Conformer1

-----------------------------------------------------------------------------------------------------

Center Atomic Atomic Coordinates (Angstroms)

Number Number Type X Y Z

------------------------------------------------------------------------------------------------------

1 6 0 0.646474 1.250456 -0.085920

2 6 0 -0.696737 1.305516 -0.288930

3 6 0 -1.542349 0.077740 -0.443751

4 6 0 -0.742290 -1.188553 -0.311563

5 6 0 0.596891 -1.191295 -0.100305

6 6 0 1.348810 0.018108 0.009589

7 1 0 1.210514 2.177752 0.018484

8 1 0 -1.217935 2.260034 -0.333993

9 1 0 -2.015646 0.100511 -1.447235

10 1 0 -1.287783 -2.129594 -0.392684

11 1 0 1.125621 -2.140918 -0.007180

12 6 0 2.830289 -0.027869 0.244366

13 1 0 3.263232 0.978545 0.259121

14 1 0 3.065644 -0.512718 1.203448

15 1 0 3.339517 -0.607666 -0.539012

16 8 0 -2.592814 0.154268 0.532831

17 1 0 -3.216465 -0.566312 0.387838

------------------------------------------------------------------------------------------------------

*E* = –347.1717552 a.u.

*p*-C_6_H_5_OHCH_3_ Conformer2

------------------------------------------------------------------------------------------------------

Center Atomic Atomic Coordinates (Angstroms)

Number Number Type X Y Z

------------------------------------------------------------------------------------------------------

1 6 0 0.645406 1.239262 -0.053417

2 6 0 -0.692417 1.304146 -0.256746

3 6 0 -1.542508 0.083101 -0.444217

4 6 0 -0.747332 -1.188425 -0.345783

5 6 0 0.596058 -1.201495 -0.130359

6 6 0 1.347207 -0.004362 0.012278

7 1 0 1.213309 2.161382 0.076309

8 1 0 -1.211029 2.260996 -0.278202

9 1 0 -2.016208 0.135270 -1.446319

10 1 0 -1.294710 -2.125502 -0.454476

11 1 0 1.120857 -2.154960 -0.061632

12 6 0 2.828896 -0.027417 0.248291

13 1 0 3.363431 0.542068 -0.525879

14 1 0 3.083885 0.432315 1.214579

15 1 0 3.217931 -1.051751 0.247152

16 8 0 -2.592072 0.140454 0.534814

17 1 0 -3.207895 -0.585814 0.384853

------------------------------------------------------------------------------------------------------

*E* = –347.1717926 a.u.
